# Development and validation of large language model rating scales for automatically transcribed psychological therapy sessions

**DOI:** 10.1038/s41598-025-14923-y

**Published:** 2025-08-12

**Authors:** Steffen T. Eberhardt, Antonia Vehlen, Jana Schaffrath, Brian Schwartz, Tobias Baur, Dominik Schiller, Tobias Hallmen, Elisabeth André, Wolfgang Lutz

**Affiliations:** 1 Department of Psychology, Trier University, Trier, Germany; 2 Chair for Human-Centered Artificial Intelligence, Augsburg University, Wissenschaftspark 25+27, 54296 Trier, Germany

**Keywords:** Natural language processing (NLP), Patient engagement, Measurement-based care (MBC), Automatized transcription, Human behaviour, Psychology, Medical research, Outcomes research, Diagnosis, Quality of life, Health care, Therapeutics, Adverse effects

## Abstract

Rating scales have shaped psychological research, but are resource-intensive and can burden participants. Large Language Models (LLMs) offer a tool to assess latent constructs in text. This study introduces *LLM rating scales*, which use LLM responses instead of human ratings. We demonstrate this approach with an LLM rating scale measuring patient engagement in therapy transcripts. Automatically transcribed videos of 1,131 sessions from 155 patients were analyzed using DISCOVER, a software framework for local multimodal human behavior analysis. Llama 3.1 8B LLM rated 120 engagement items, averaging the top eight into a total score. Psychometric evaluation showed a normal distribution, strong reliability (ω = 0.953), and acceptable fit (CFI = 0.968, SRMR = 0.022), except RMSEA = 0.108. Validity was supported by significant correlations with engagement determinants (e.g., motivation, *r* = .413), processes (e.g., between-session efforts, *r* = .390), and outcomes (e.g., symptoms, *r* = − .304). Results remained robust across bootstrap resampling and cross-validation, accounting for nested data. The LLM rating scale exhibited strong psychometric properties, demonstrating the potential of the approach as an assessment tool. Importantly, this automated approach uses interpretable items, ensuring clear understanding of measured constructs, while supporting local implementation and protecting confidential data.

**Supplementary Information:**

The online version contains supplementary material available at 10.1038/s41598-025-14923-y.

## Introduction

Rating scales and other measurement instruments have played a central role in assessing psychological constructs, enabling researchers and practitioners to quantify behaviors, emotions, and therapeutic processes. In clinical psychology, these tools are essential for tracking patient progress, evaluating treatment efficacy, and ensuring evidence-based care^1^. Continuous measurement throughout treatment allows practitioners to refine their interventions and align them with patients’ evolving needs. Traditional rating scales, such as self-report and observer-based instruments, have yielded significant advances in psychological assessment. Approaches such as routine outcome monitoring (ROM)^2^, measurement-based care (MBC)^3^, and feedback-informed therapy (FIT)^4^ exemplify the practical achievements enabled by these measures. By systematically incorporating measurement into therapy, these approaches have demonstrated significant improvements in symptom reduction, reduced dropout rates, and better outcomes for not-on-track cases^5^.

Despite their benefits, traditional methods are not without limitations. Self-report instruments are prone to response biases such as social desirability and recall effects, which can compromise the validity of the results^6^. Observer-based ratings, while valuable, require significant resources, including intensive training, careful rater selection, and the time-consuming process of conducting and reviewing ratings to ensure reliability^7^. Additionally, the response burden associated with frequent assessments can hinder patients’ willingness to participate, limiting the granularity of data collected^8^.

Recent advancements in Natural Language Processing (NLP) and Large Language Models (LLMs) present promising possibilities for addressing some of the limitations of traditional measures^9^. NLP technologies have made significant advances in text analysis, enabling researchers to extract nuanced information from large amounts of data. Given that psychological therapies are predominantly conversational and language-based, these tools may offer valuable new ways to study therapeutic processes and outcomes. For example, NLP has been used to analyze therapy session transcripts to predict patient distress^10^, study emotional coherence^11^, and measure emotional tone^12,13^. Furthermore, topic modeling, another NLP technique, has been applied to assess therapeutic alliance and symptom severity^14^, while machine learning models incorporating NLP have been employed to evaluate multicultural orientation in therapy^15^.

Beyond text-based applications, video analysis has emerged as another powerful tool for automated measurement in clinical psychology. Deep learning methods have been used to assess non-verbal emotional expressions in psychological therapies, capturing aspects of the therapeutic interaction that are difficult to measure using traditional methods^16^. Notably, advancements in automated transcription allow audio data from audio-visual recordings to be converted into text, enabling the seamless integration of video analysis and NLP approaches. These developments underscore the potential of combining modalities to gain a more comprehensive understanding of therapeutic processes^17^.

With the growing use of NLP and LLMs as measurement instruments in clinical psychology, it is becoming increasingly important to apply established psychometric principles to ensure the objectivity, reliability, and validity of such automated measures. To address this need, our study applies classical test theory and scale construction principles to develop and evaluate automated measures based on LLMs. Specifically, we propose the creation of an LLM rating scale approach. An LLM rating scale is a psychometric tool for measuring latent constructs through the analysis of text data. It mirrors traditional rating scales by using a structured set of items, assigning numerical values to responses, and ensuring psychometric evaluation for reliability and validity. However, instead of human ratings, it uses LLM-generated responses derived through prompts in combination with text inputs such as therapy transcripts, session documentation, or other case records.

To test the utility of this approach, the study focuses on the construct of patient engagement, a concept critical to the success of psychological therapies. Engagement is a multifaceted construct encompassing both motivational and relational aspects of the therapeutic process^18^. It reflects the extent to which patients are invested in and connected to therapy, including their willingness to actively participate, their relational bond with the therapist, and their alignment with therapeutic goals. Holdsworth et al.’s *Model of Client Engagement in Psychotherapy*^18^ provides a robust theoretical framework for conceptualizing engagement. This model differentiates between engagement determinants (e.g., client motivation, therapeutic relationship), processes (e.g., attendance, within- and between-session efforts), and outcomes (e.g., treatment success). Building on this foundation, this study develops and evaluates the Large Language Model Engagement Assessment in Psychological Therapies (LLEAP), an LLM rating scale designed to automatically measure engagement by analyzing therapy session transcripts.

In addition, the study addresses practical challenges in clinical psychological research. It explores the automation of transcription processes to reduce resource demands and demonstrates how LLMs can be implemented locally to ensure confidentiality of sensitive patient data, overcoming privacy concerns associated with cloud-based LLM solutions (e.g., ChatGPT). While the primary focus is on psychological therapies, the methodology has broader applications in psychological research, particularly in areas reliant on conversational or text-based data.

The objectives of the study are threefold: First, it aims to create a semi-automated pipeline that integrates transcription, item generation, and item selection. Second, the study seeks to develop an LLM rating scale (i.e., LLEAP), designed to automate the measurement of patient engagement in psychological therapies. Finally, the psychometric properties of this LLM-based scale will be evaluated, focusing on key metrics such as reliability, model fit, and validity. In line with these objectives, we have formulated specific hypotheses. We anticipate that the LLM rating scale will demonstrate acceptable reliability (H1). Furthermore, we expect the scale to exhibit an acceptable model fit (H2). Lastly, we predict that the LLM-based measure of patient engagement will establish validity by showing significant correlations with key determinants (i.e., motivation, alliance), processes (i.e., between- and within-session effort), and outcomes (i.e., symptom outcome) of engagement (H3).

## Method

The development and evaluation of the LLM rating scale followed a structured, multi-stage process. First, psychological therapy sessions were automatically transcribed, diarized, and segmented using a local pipeline, ensuring privacy-preserving and scalable data preparation. These segmented transcripts were then paired with an initial pool of 120 theoretically derived items, which were rated by a local LLM. Next, we applied a psychometric selection pipeline to identify a subset of items that met distributional, reliability, and validity criteria. To avoid inflated estimates due to item selection on the same data, we employed two complementary strategies: (1) repeated 3-fold cross-validation to ensure strict independence between item selection (in training folds) and psychometric evaluation (in test folds), and (2) bootstrap optimism correction, which adjusts performance metrics based on differences between bootstrap samples and the original data. These approaches allowed us to assess the generalizability and robustness of the final scale under different methodological constraints, accounting for potential overfitting.

### Patients and therapists

The sample included 1,131 session transcripts from 155 patients (*M*_age_ = 36.37 years, *SD*_*age*_ = 13.95; 61.9% female). Patients were included in the study if (a) they and their therapists gave informed consent to the usage of their data for research purposes and (b) they had at least four transcribed sessions. No restrictions were made based on demographic variables or psychopathology. Patients had, on average, 7.3 (*SD* = 3.03) transcribed sessions. Table S1 shows the diagnoses based on the Structured Clinical Interview for DSM-IV and DSM-5^19,20^. The most common diagnosis was depressive disorder (52.6%), followed by panic/agoraphobia (13.0%) and adjustment disorder (11.7%). All therapy sessions were conducted in German. The patients were treated by 95 therapists (*M*_*age*_ = 28.01 years, *SD*_*age*_ = 4.20, 84.6% female). They had a master’s degree in psychology, were in a 3–5-year psychotherapy training program, and had at least 1.5 years of clinical experience. The mean number of patients per therapist was 2.5 (*SD* = 1.96).

### Ethics declaration

All methods were carried out in accordance with relevant guidelines and regulations. All procedures in this study complied with the ethical standards of the relevant national and institutional committees on human experimentation and with the Helsinki Declaration of 1975 and its later amendments. The research project received approval from the ethics committee of Trier University. Written, informed consent allowing data to be used for research purposes was obtained from all participants. Protecting the confidentiality and privacy of patient data was a core objective of this study. All therapy session transcripts remained on secure institutional servers and were never transmitted to third-party providers or cloud-based services. Both the speech-to-text transcription and LLM analyses were conducted locally within a secure computing environment. While the transcription was performed using locally executed speech-to-text models, the LLM analyses were conducted using a self-hosted instance of an open-source LLM deployed via the Ollama framework. Importantly, the LLM was used exclusively for inference, meaning that patient data were input to generate responses, but the model itself was neither modified nor updated in any way. No patient data were stored by the model or incorporated into its internal parameters. This setup ensured that all data processing was fully local and that patient confidentiality was maintained throughout the analysis pipeline.

### Treatment and supervision

Between 2016 and 2024, personalized integrative cognitive-behavioral therapies (CBT) were conducted at a university outpatient training and research clinic in Southwest Germany. Therapists had supervision and training in manualized treatments and transtheoretical concepts. The supervisors were licensed cognitive behavior therapists with a minimum of five years of clinical experience and advanced training in supervision, maintaining ongoing professional development and continuous education. Patients received individual therapy sessions that lasted 50 min and took place weekly.

### Measures

Patients completed questionnaire batteries at intake and after treatment termination, after every fifth session, and short scales before and after each session as part of routine process and outcome monitoring at the outpatient clinic. Therapists answered brief questionnaires at the end of each session. The R package *missForest* v1.5 was used to impute missing values^21^.

### Measures of engagement determinants

Measures of engagement determinants included motivation, assessed at session 15, and alliance, assessed after each session.

#### Motivation

Patient motivation was assessed after session 15 using the motivation subscale of the Assessment for Signal Clients (ASC)^22^. The scale is rated on a five-point Likert scale, ranging from 1 *(does not apply at all)* to 5 *(fully applies)*. Session 15 was selected, because this session most closely aligned with the average transcribed session (*M* = 14.01) across all patients. The motivation subscale consists of nine items, asking patients to reflect on their feelings about treatment during the past week (e.g., “I wonder what I am doing in therapy; actually I find it boring.”). Scores were averaged and inverted in a way that higher scores indicated higher patient motivation for therapy. Reliability was good (α = 0.83).

#### Alliance

Alliance was measured from both the patient and therapist perspective using a short version of the Bern Post-Session Report (BPSR)^23^ after each session. The items were assessed on a seven-point Likert scale ranging from − 3 *(not at all)* to 3 *(yes*,* exactly)*. They were averaged to obtain the final scores for each subscale. In the current study, reliability was good: α = 0.86 for patient-rated alliance (4 items) and α = 0.87 for therapist-rated alliance (3 items) at session 15.

### Measures of engagement processes

Engagement processes included within-session effort (problem coping) and between-session effort (willingness) and were assessed after each session using the short version of the BPSR^23^. The within-session effort was rated by the patient, whereas the between-session effort was rated by the therapist.

#### Within-session effort

Patient-rated problem coping was used as a proxy for within-session effort. Problem coping was measured after each session using three items from the BPSR^23^ evaluating whether patients felt more confident solving their problems independently, had a clearer understanding of their goals, and felt better able to handle previously overwhelming situations. Responses were rated on a seven-point Likert scale ranging from − 3 *(not at all)* to 3 *(exactly)* and averaged to calculate a final score. In the present study, problem coping (3 items) reliability was excellent (α = 0.91) at session 15.

#### Between-session effort

Patients’ willingness to work on their problems between sessions was evaluated by the therapist after each session using a single item from the BPSR^23^. The item, “I have the impression that the patient works intensively between sessions on what we have discussed in therapy” (translated from German), is rated on a seven-point Likert scale ranging from − 3 *(not true at all)* to 3 *(exactly right)*. While relying on a single item, its content is highly consistent with between-session effort as described in Holdsworth et al.’s Model of Client Engagement^18^.

### Measure of engagement outcome

Symptom severity was assessed at session 25 as a measure of treatment outcome. This session was chosen because it occurred approximately 10 sessions after the average transcribed session (*M* = 14.01), providing sufficient time for patients to experience change and for treatment effects to manifest. The Outcome Questionnaire-30 (OQ-30)^24^ was used to evaluate symptom severity. This 30-item self-report measure assesses various aspects of psychological functioning, including subjective complaints, interpersonal relationships, and fulfillment of social roles. Patients responded on a five-point Likert scale ranging from 0 *(never)* to 4 *(almost always)*. Higher overall mean scores indicate greater psychological distress. In the current study, reliability of the OQ-30 was excellent (α = 0.95).

### Software framework

For our study, we used DISCOVER^25^, an open-source software framework for multimodal human behavior analysis. It facilitates computational human behavioral analysis by supporting diverse data types and workflows for researchers without extensive technical expertise. DISCOVER integrates NOVA^26^, a graphical interface for visualizing data streams and annotations, and machine learning models for audio-visual data processing. NOVA enhances the standard annotation process with developments from contemporary research fields, such as Cooperative Machine Learning and Explainable Artificial Intelligence. It gives annotators access to automated model training, prediction functionalities, and sophisticated explanation algorithms via its user interface.

### Transcript Preparation

The therapy sessions were video-recorded using Telycam TLC-700-S-R cameras and Beyerdynamic BM 32 W microphones. The videos were used for automatized transcription.

#### Video screening

The video screening process prioritized audio quality, essential for generating reliable session transcripts. Trained assistants used a standardized protocol to ensure consistent assessment across recordings and signed confidentiality agreements. The screening confirmed that audio was clear, uninterrupted, with no significant technical issues. Assistants also documented the timing of therapeutic conversations, noting start and end times, and who initiated the session. Speech samples were selected by identifying the longest continuous sequences (at least 10 s) for both patient and therapist, with exact start and end times recorded. These samples enhanced the accuracy of automated speaker diarization. Only recordings with exactly two speakers, patient and therapist, were included in the study.

#### Transcription

For transcription, we used DISCOVER, which integrates WhisperX^27^ to transcribe session audio with timestamps. WhisperX, based on the Whisper model^28^, was configured with the “large-v3” model in “segment” alignment mode for coherent audio segmentation. The language was set to German, with a batch size of 16 for simultaneous processing, and computations were performed in float32 precision. To evaluate transcription quality, we compared WhisperX-generated transcripts with manually transcribed reference versions from 342 psychological therapy sessions. In the automatic transcripts, speaker diarization was performed using the method described below. In contrast, the manual transcripts were produced by human transcribers who had access to both audio and video material and could therefore directly assign each utterance to either the patient or the therapist. For comparison, all transcripts were separated into patient and therapist statements. The *process_words* function from the *jiwer* v4.0.0 Python package^29^ was used to align manually and automatically transcribed texts and to annotate substitutions, insertions, and deletions. The word error rate (WER), calculated as the proportion of these discrepancies relative to the total number of words, was computed for each sentence and then averaged across transcripts, yielding a mean WER of 26.76 (*SD* = 3.45). To assess semantic similarity, we used BGE-M3^30^ embeddings to derive sentence representations and calculated the average cosine similarity between aligned sentence pairs. This analysis yielded a high mean similarity score of 0.90 (*SD* < 0.01), on a scale from 0 *(no similarity)* to 1 *(identical meaning)*. These results suggest that, despite some transcription errors, the core content of therapeutic conversations was preserved, supporting the adequacy of WhisperX transcriptions for subsequent NLP applications such as LLM rating scales.

#### Speaker diarization

Speaker diarization distinguished between patient and therapist in the audio recordings. This step was necessary because each session was captured using a single microphone, resulting in one unified audio recording per session. While WhisperX handled transcription, DISCOVER used SpeechBrain^31,32^ for diarization. This open-source PyTorch toolkit computed embeddings of voiced segments in the audio signal which are then clustered by the ‘finch’ clustering method using the ‘cosine’ metric. A reference-based oracle approach assigned these clusters to individual speakers (‘therapist’ or ‘patient’). Human screeners documented who initiated the conversation and manually selected reference samples, each at least 10 seconds long, ensuring accurate and representative voice samples for each speaker.

#### Transcript segmentation

Due to LLM token limits, transcripts were split into 4,574 segments of up to 2,000 tokens to ensure accurate processing. In NLP, a token is a small unit of text used for processing, such as a word, subword, or punctuation mark. A custom function split transcripts into intact sentences, adding them sequentially until the token limit was reached. The 2,000-token limit allowed room for additional prompt-related inputs, while staying within the LLM’s capacity.

### Scale development

We applied basic scale construction principles to the development of the LLM-based rating scale^33^.

#### Item generation

The item pool for measuring patient engagement in psychological therapies was developed using a deductive approach^33^, based on Holdsworth et al.‘s Model of Client Engagement^18^. Definitions and components of engagement were derived from this model to ensure a theory-driven basis for item development. ChatGPT 4o^34^ was used to facilitate the item generation process. It was prompted with a reference to the Model of Client Engagement, relying on its pre-existing knowledge of the model to generate items aligned with its conceptual structure. Since the LLM analyzes therapy transcripts without direct access to the patient’s internal experiences, all items were formulated from an observer perspective.

To specify the format, ChatGPT was provided with the following example prompt: “Please rate how motivated the patient is to engage in therapy on a scale from 0 (not motivated to engage at all) to 100 (highly motivated to engage).” It was then instructed to generate similar items for the different components of engagement. To ensure comprehensive coverage of nuanced engagement facets, it was also prompted to generate lists of synonyms and related terms, which were subsequently used to create additional prompts. The chat history documenting this process can be found in the supplemental material. To ensure that the LLM responded with a numeric rating that could be easily extracted for further analysis, each prompt included the following instruction, specifying the use of JavaScript Object Notation (JSON): “Please respond in JSON format: {‘rating’: [insert rating between 0-100 here]}. Do not write anything else.” The final item pool consisted of 120 items and is available in Table S8 in the supplemental material.

#### Large language model application

We used DISCOVER^25^, with its integrated LLMs, to process transcripts of psychological therapy sessions. To ensure privacy and confidentiality, DISCOVER was configured with a self-hosted LLM using Ollama v0.3.3^35^. The selected model, Llama 3.1 8B^36^ was deployed on a computer equipped with a 13th generation Intel Core i9-13900 K processor, 64 GB of RAM, and an NVIDIA GeForce RTX 4090 GPU. The model operated as a classifier, guided by the following system prompt: ‘You are a classifier that assigns ratings to text segments.’ The data description provided to the model specified: ‘The texts or text segments contain patient (P) and therapist (T) statements in a psychotherapy transcript. The patient statements are preceded by a “P:” and the therapist statements by a “T:“.’ This structure allowed the LLM to differentiate between statement types and better understand the interaction context. Model parameters included top_k set to 50, top_p to 0.95, and temperature to 0, ensuring deterministic and consistent outputs by eliminating randomness. Prompts were paired with each transcript segment, ensuring the LLM processed every prompt independently. Each prompt was presented separately to avoid influence from prior prompts and prevent overloading the LLM, reducing the risk of hallucinations or errors.

#### Item selection

The selection of items followed a systematic, automated pipeline designed to ensure psychometric quality and theoretical alignment with the construct of interest. The process, implemented using custom R functions, allowed repetition in bootstrap and repeated k-fold cross-validation procedures. The pipeline consisted of two main stages: pre-selection and final selection. During *pre-selection*, items were screened to eliminate those failing to meet minimum psychometric standards. Distribution properties were assessed using the Kolmogorov-Smirnov (K-S) test for normality, alongside skewness, kurtosis, and standard deviation thresholds. Items meeting at least two criteria, such as skewness and kurtosis within [− 1,1] and standard deviation ≥ 7, were retained. Difficulty indices, calculated as the ratio of mean score to maximum possible score, were used to exclude items outside the range of 0.30 to 0.85. Item-total correlations were computed to ensure that each item meaningfully contributed to overall reliability, with only items exceeding a threshold of 0.70 retained. Finally, a single-factor exploratory factor analysis (EFA) identified items with strong alignment to the construct, retaining those with factor loadings ≥ 0.70. During *final selection*, the remaining items were evaluated based on their correlations with validation scales. A composite score was calculated for each item across all validation measures, and items were ranked by these scores. The top eight correlating items were selected for the final scale, emphasizing the reduction of item numbers to enhance both parsimony and computational efficiency, as fewer items lower the demands of processing large item pools using LLMs.

### Scale evaluation

After automatically selecting items for the LLEAP scale, its psychometric properties were evaluated in four key areas: scale distribution, reliability, model fit, and validity. Scale evaluation was conducted on the original sample and its stability and generalizability were further examined using bootstrap resampling and repeated 3-fold cross-validation.

#### Evaluation of scale distribution using descriptive statistics

The LLEAP score was calculated as the mean of the selected items and its distribution was examined at the patient level using descriptive statistics. The LLEAP score was aggregated for each patient, and descriptive statistics (e.g., *M*, *SD*, skewness, and kurtosis) were calculated. Normality was assessed using K-S tests. A histogram was created to visualize the LLEAP score distribution. This analysis was conducted on the original sample.

#### Evaluation of reliability and model fit using confirmatory factor analysis

Reliability and model fit of the LLEAP scale were assessed using Multilevel Confirmatory Factor Analysis (MCFA) to address the hierarchical data structure. MCFA was conducted using the *lavaan* package in R, with the *sem* v.0.6–19 function^37^. Engagement was modeled as a single latent factor, with the cluster argument accounting for session nesting within patients. Reliability estimates, including Cronbach’s α, McDonald’s ω, and Average Variance Extracted (AVE), were calculated using the *semTools* v0.5-6 reliability function^38^. Cronbach’s alpha provided a traditional measure of internal consistency (α > 0.70). McDonald’s omega (ω > 0.70) was also calculated for a more robust reliability estimate. To test hypothesis H1, we primarily relied on results obtained from the independent test folds in the cross-validation procedure, as this represents the most rigorous assessment of scale reliability, unaffected by overfitting due to item selection. AVE values above 0.50 indicated that the factor explained more variance than measurement error. Model fit was evaluated using Root Mean Square Error of Approximation (RMSEA), Comparative Fit Index (CFI), Tucker-Lewis Index (TLI), and Standardized Root Mean Square Residual (SRMR). Acceptable fit thresholds included RMSEA ≤ 0.08, CFI > 0.90, TLI > 0.90, and SRMR < 0.08. These analyses were performed on the original sample, bootstrap samples, and test folds. To test hypothesis H2, we focused on the model fit indices derived from the cross-validated test folds, as they reflect performance in strictly independent evaluation sets and therefore provide a conservative and unbiased estimate of model fit.

#### Evaluation of validity using correlations and multilevel modeling

The validity of the LLEAP scale was assessed using correlation analyses and multilevel modeling (MLM). Zero-order Pearson correlations were calculated between the LLEAP score and validation scales at the patient level to evaluate the strength of relationships. Following Cohen’s conventions (*r* = .10 small, *r* = .30 medium, *r* = .50 large)^39^, correlations were interpreted for their effect sizes. These were calculated in the original sample, bootstrap samples, and test folds.

MLM was conducted using the *lme4* v1.1-35.5 package in R, with the *lmer* function^40^. For session-level validation scales (e.g., alliance, within-session effort, between-session effort), models accounted for the nested structure of sessions within patients. To capture within-patient variability, the LLEAP score was patient mean-centered and included as a Level 1 predictor, while the patient mean LLEAP score was included as a Level 2 predictor to assess between-patient effects (Equation S1). Cluster-robust standard errors were calculated using the *lmerTest* v3.1-3 package to account for clustering within therapists^41^. For patient-level validation scales (e.g., motivation, symptom outcome), multilevel models analyzed the relationship between the patient mean LLEAP score and validation scales, accounting for variability attributable to differences between therapists (Equation S2). Detailed model formulas and descriptions can be found in the supplement.

#### Bootstrap optimism correction and repeated k-folds cross-validation

To validate the psychometric properties of the scale and ensure its generalizability, we employed two complementary validation techniques: bootstrap optimism correction and repeated k-fold cross-validation, each with distinct strengths and limitations. Bootstrap optimism correction provides an estimate of overfitting by comparing performance on resampled datasets with the original dataset. However, it does not fully replicate realistic evaluation scenarios due to the partial overlap between resampled and original data. In contrast, repeated k-fold cross-validation better reflects practical test conditions by systematically separating training and test sets. This approach, however, reduces evaluation power as the test fold contains only a fraction of the total sample (one-third in 3-fold cross-validation), leading to increased variability, particularly for smaller datasets.

In the bootstrap procedure, 1,000 resampled datasets were generated with replacement. Optimism was calculated as the difference in performance metrics, including correlation matrices, confidence intervals, reliability estimates, and model fit indices, between the bootstrap sample and the original dataset^42^. A strict optimism correction was applied by penalizing the original performance metrics with the absolute value of the optimism, ensuring conservative corrected estimates. Item selection frequencies across all iterations were tracked to evaluate their consistency and robustness.

Repeated k-fold cross-validation was performed using three folds repeated 20 times, stratified at the patient level to prevent data leakage. The training folds were used for item selection and the test folds were used for scale evaluation. Repetition reduced variability caused by specific data partitions, providing more stable and generalizable performance estimates, particularly for smaller datasets^43^. Performance metrics, including correlations, model fit, and reliability estimates, were aggregated across test folds to compute mean performance metrics and confidence intervals. Item selection frequencies in the training folds were also counted to assess their stability. The cross-validation results were the primary basis for hypothesis testing, as they represented the more rigorous test of the hypotheses, as cross-validation is the more conservative validation strategy due to the strict separation of training and test data. To test hypothesis H3, we used the aggregated 95% confidence intervals of the MLMs derived from the cross-validation analyses to evaluate associations between LLEAP and the validation scales as the MLMs better account for the hierarchical structure of the data (e.g., sessions nested within patients) than zero-order correlations.

## Results

Using the LLM to process the 120 engagement items in combination with 4,574 segments from 1,131 transcripts took approximately 60 h. The eight items automatically selected for the LLEAP (Table 1) were consistently identified as the most frequently selected items across 1,000 bootstrap samples and 20 repetitions of 3-fold cross-validation.

**Table 1:** Items of the large language model engagement assessment in psychological therapies (LLEAP). Each prompt concluded with the instruction: ‘Please respond in JSON format: {“rating”: [insert rating between 0-100 here]}. Do not write anything else.’

#	Facet	Prompt text
1	Effort Betw. Sessions	Please rate how much effort the patient puts into therapeutic activities between sessions on a scale from 0 (no effort) to 100 (maximum effort).
2	Homework Completion	Please rate how consistent the patient is in completing assigned therapy homework on a scale from 0 (never completes) to 100 (always completes).
3	Motivated for Homework	Please rate how motivated the patient is to complete their therapy homework on a scale from 0 (not motivated at all) to 100 (highly motivated).
4	Accountable for Homework	Please rate how accountable the patient is for their homework and practice activities on a scale from 0 (not accountable at all) to 100 (fully accountable).
5	Participation in Exercises	Please rate how often the patient participates in therapeutic exercises or activities assigned during sessions on a scale from 0 (never participates) to 100 (always participates).
6	Engages with Techniques	Please rate how actively the patient engages with therapeutic techniques outside of sessions on a scale from 0 (not engaged at all) to 100 (fully engaged).
7	Behavioral Changes	Please rate how well the patient implements behavioral changes discussed in therapy on a scale from 0 (not at all) to 100 (very well).
8	Readiness for Sessions	Please rate how ready the patient is to engage in each therapy session on a scale from 0 (not ready at all) to 100 (completely ready).

### Scale distribution

**Fig. 1:**
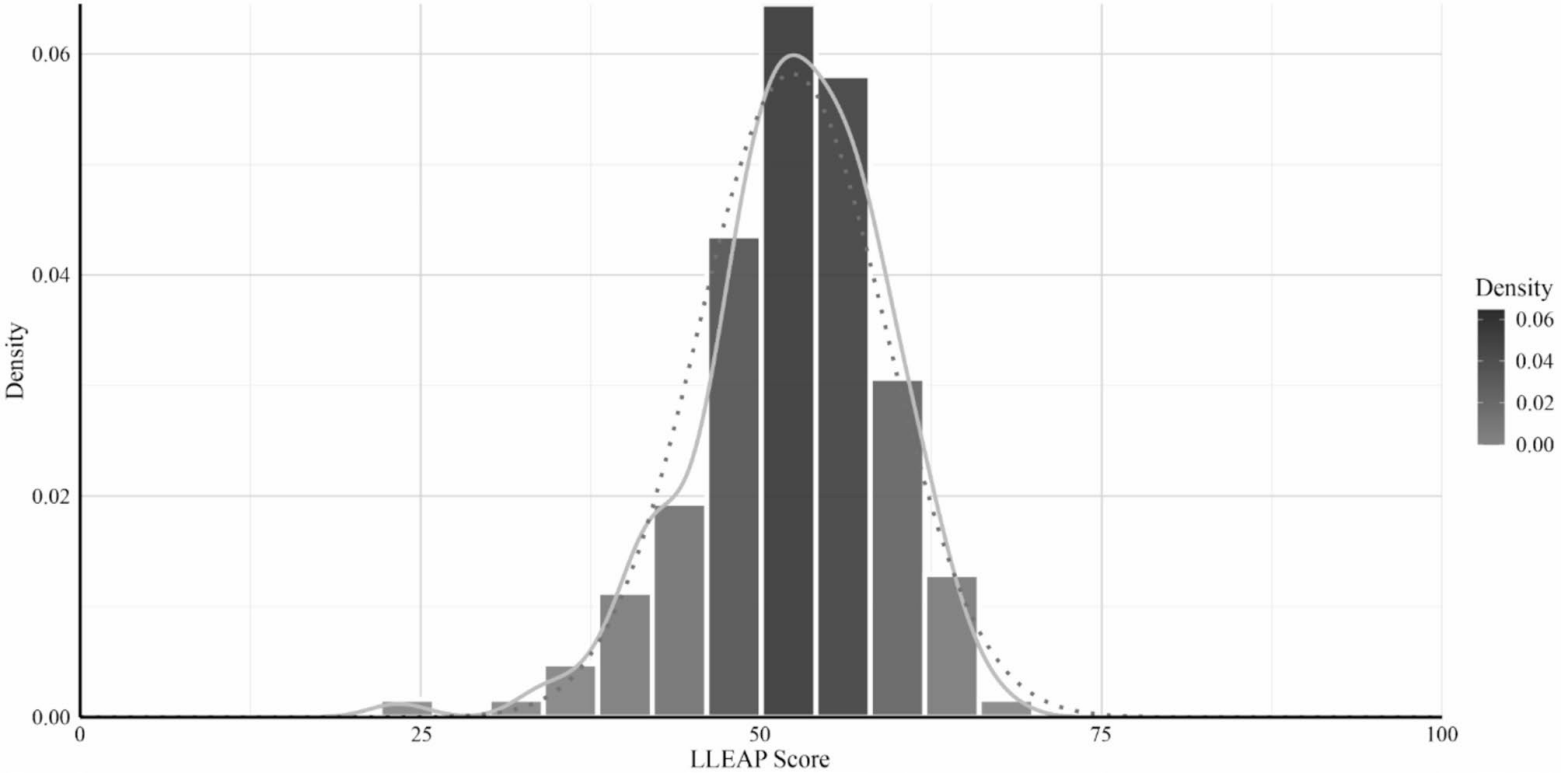
Histogram of LLEAP with density and normal curve in the original sample of *N*_P_ = 155 patients. The solid line represents the normal curve and the dotted line represents the density curve.

The LLEAP scale had a mean score of *M* = 52.27 (*SD* = 6.84) in the original sample (*Np* = 155). The distribution showed slight negative skewness (− 0.73), indicating a small tail to the left, and a kurtosis value of 1.35, suggesting a moderately peaked distribution. The K-S test value was 0.06 (*p* = .72), indicating no significant deviation from normality. The distribution of LLEAP scores is visualized in Fig. 1. Further descriptive statistics are available in Table S2 in the supplemental material.

### Reliability and model fit

The results for reliability and model fit are summarized in Table 2. In line with hypothesis H1, the reliability analysis based on the average values across the repeated test folds indicated strong internal consistency, with α = 0.946 and ω = 0.947. The AVE was 0.691, exceeding the recommended threshold of 0.500. With respect to hypothesis H2, model fit indices indicated mixed results across validation methods. In the original sample, SRMR was 0.022, indicating a good fit (< 0.08), while CFI (0.968) and TLI (0.956) exceeded recommended thresholds (> 0.90). However, RMSEA was 0.108 and above the threshold for acceptable fit (< 0.08). After bootstrap optimism correction and cross-validation, the fit indices declined slightly (e.g., cross-validated RMSEA = 0.135, CFI = 0.945, TLI = 0.923, SRMR = 0.033), but remained within acceptable ranges for SRMR, CFI, and TLI.

**Table 2:** Model fit and reliability of LLEAP using different validation methods.* N*_*P*_ = 155 for original and optimism corrected values. Cross-validated values used ∼52 patients per fold, with all patients contributing across folds. Model fit indices: CFI and TLI (> 0.90 acceptable), RMSEA (< 0.08 acceptable), SRMR (< 0.08 good). Reliability: α (Cronbach’s alpha; > 0.70 acceptable, > 0.90 excellent) measures internal consistency; ω (McDonald’s omega; > 0.70 acceptable, > 0.90 excellent) provides a robust reliability estimate; AVE (Average Variance Extracted) reflects the proportion of variance explained by the latent construct (> 0.50 good). Validation methods: Original Sample = estimates without corrections; Optimism Corrected = adjusted for overfitting via bootstrapping; Cross-Validated = derived from repeated cross-validation to assess generalizability.

Validation	Model Fit				Reliability		
	CFI	TLI	RMSEA	SRMR	α	ω	AVE
Original Sample	0.968	0.956	0.108	0.022	0.952	0.953	0.715
Optimism Corrected	0.964	0.949	0.114	0.024	0.952	0.952	0.713
Cross-Validated	0.945	0.923	0.135	0.033	0.946	0.947	0.691

### Validity

**Fig. 2:**
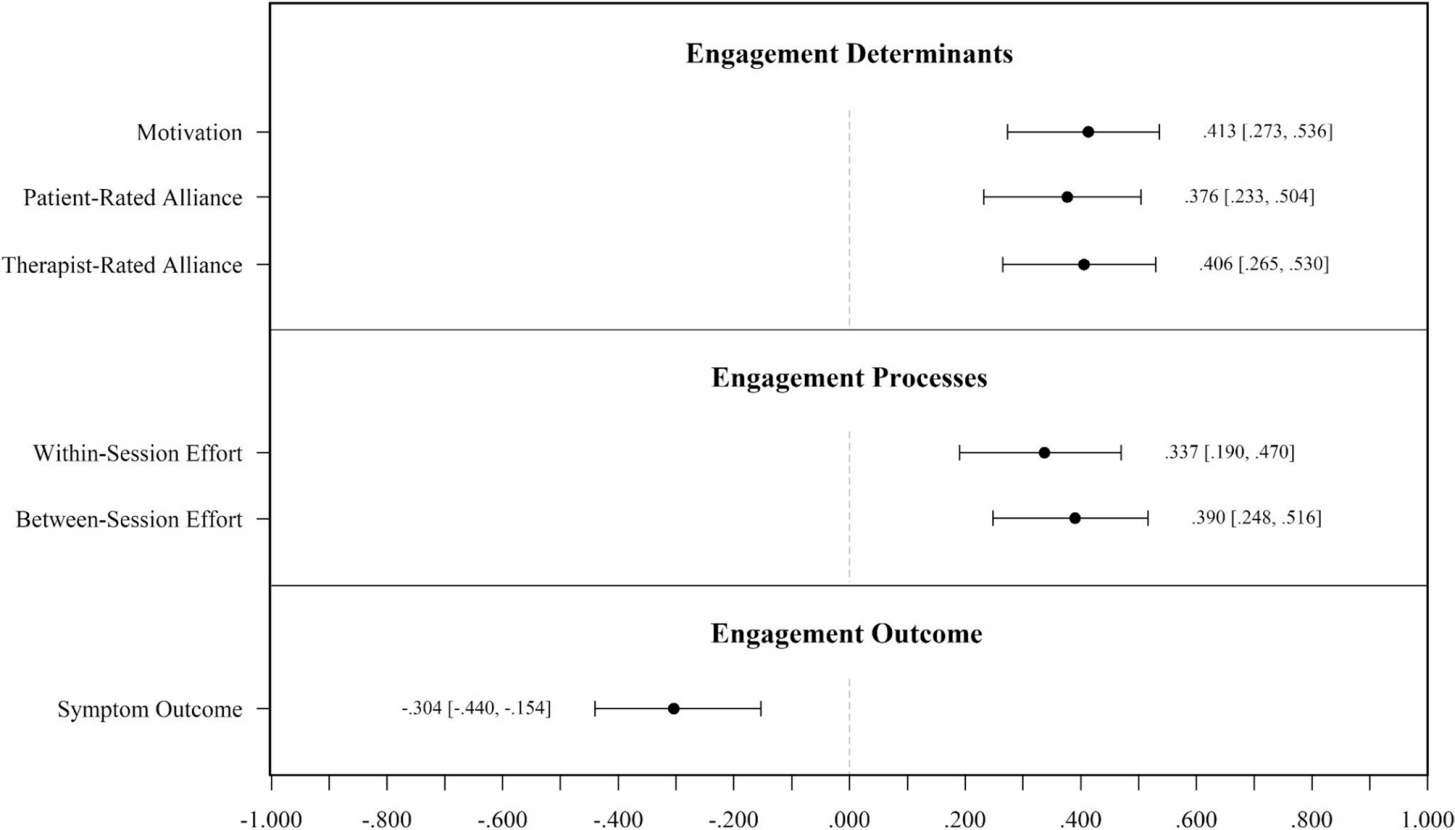
Between-patient correlations of LLEAP with the validation scales in the original sample of *N*_P_ = 155 patients. Correlations among the validation scales, bootstrap optimism-corrected correlations, and cross-validated correlations can be found in the Supplementary Tables S3–S5.

Figure 2 visualizes the correlations between LLEAP and the validation scales from the original sample. Complete correlation matrices, including correlations among validation scales, are provided in the supplemental material for the original sample (Table S3), bootstrap optimism correction (Table S4), and cross-validation (Table S5). Table S6 compares these correlations across validation methods.

To test hypothesis H3, MLMs were conducted, which accounted for the nested data structure and applied cross-validation to ensure conservative estimates. Results of these analyses, shown in Table S7, revealed that all effects relevant to hypothesis H3 were statistically significant in the test folds, with 95% confidence intervals not including zero. For validation scales measured at the session level (i.e., alliance, within-session effort, between-session effort), both patient mean LLEAP scores (Level 2 predictors) and patient mean-centered LLEAP scores (Level 1 predictors) were significant.

Significant between-patient effects showed that patients with higher overall engagement scores reported greater motivation for therapy (b = 0.174, 95% CI [0.059, 0.289]), had stronger patient-reported alliance (b = 0.167, 95% CI [0.007, 0.326]) and therapist-reported alliance (b = 0.193, 95% CI [0.042, 0.344]), more frequent within-session problem coping experiences (b = 0.305, 95% CI [0.055, 0.555]), and greater between-session effort as reported by their therapists (b = 0.278, 95% CI [0.065, 0.490]). These patients also reported fewer symptoms later in therapy (i.e., after session 25; b = –0.177, 95% CI [–0.344, –0.009]). Within-patient effects, reflecting session-level dynamics, indicated that sessions with higher engagement than a patient’s typical level were associated with better patient-reported alliance (b = 0.041, 95% CI [0.008, 0.073]) and more problem coping experiences in these sessions (b = 0.121, 95% CI [0.036, 0.205]), while therapists noted greater between-session efforts (b = 0.094, 95% CI [0.002, 0.187]).

## Discussion

The primary aim of this study was to develop and evaluate a rating scale that uses ratings from LLMs instead of traditional human responses (i.e., LLM rating scale). Specifically, we sought to explore whether classical test theory and established principles of scale construction could be applied to create an LLM rating scale for measuring patient engagement in psychological therapies. To achieve this, we developed a semi-automated pipeline for transcript preparation, item generation, and item selection and then evaluated the psychometric properties of the resulting LLM rating scale. Our findings provide robust support for all our hypotheses. The results indicate that the LLM-based scale, LLEAP, demonstrates strong psychometric properties, including high reliability, acceptable model fit, and meaningful correlations with established and theoretically-derived engagement constructs.

LLEAP demonstrated high reliability, with Cronbach’s α (0.952) and McDonald’s ω (0.953) both exceeding 0.90, indicating excellent internal consistency. The scale achieved strong model fit according to several criteria, including CFI (0.968) and TLI (0.956), which both exceeded the threshold of 0.90, and SRMR (0.022), which is well below the recommended cutoff of 0.08. In contrast, the RMSEA (0.108) exceeded the recommended threshold of 0.08, suggesting some residual misfit in the model. However, a simulation study suggests that RMSEA tends to overestimate misfit in models with low degrees of freedom and moderate sample sizes, even when overall fit is acceptable^44^. Despite this, LLEAP exhibited desirable distributional properties, being normally distributed and containing no missing values.

The correlations between LLEAP and validation scales provide strong evidence for its validity, revealing meaningful relationships with key constructs of engagement according to Holdsworth et al.’s Model of Client Engagement in Psychotherapy^18^. LLEAP demonstrated moderate positive correlations with patient-rated measures, such as motivation (*r* = .413) and patient-rated alliance (*r* = .376), as well as engagement processes like within-session efforts (*r* = .337). These findings highlight that LLEAP aligns well with self-reported engagement characteristics, such as clarity about therapy goals and belief in its usefulness. Similarly, LLEAP showed meaningful correlations with therapist-rated scales, including between-session effort (*r* = .390) and therapist-rated alliance (*r* = .406). Therapist-rated between-session effort reflects patients’ behaviors beyond therapy sessions, such as the effort patients invest in applying therapeutic strategies between sessions.

The relationships among the validation scales provide further context for interpreting LLEAP’s performance. Correlations among patient-rated measures, such as between motivation and patient-rated alliance (*r* = .606), tend to be stronger, reflecting shared method effects and a consistent self-report perspective. Similarly, therapist-rated scales, such as therapist-rated alliance and between-session effort (*r* = .669), demonstrate strong relationships, indicating alignment within the therapist perspective. In contrast, correlations between patient-rated and therapist-rated measures, such as patient-rated alliance and therapist-rated alliance (*r* = .416) or motivation and between-session effort (*r* = .326), are weaker, reflecting the distinctiveness of these perspectives. Notably, LLEAP’s correlations with both patient-rated and therapist-rated measures (e.g., *r* = .376 with motivation and *r* = .390 with between-session effort) are similar in magnitude to these cross-perspective relationships. For example, the correlation between LLEAP and therapist-rated alliance (*r* = .406) is comparable to the correlation between therapist-rated alliance and patient-rated alliance (*r* = .416). Apart from this, LLEAP’s correlations with engagement outcomes such as symptom severity (*r* = − .304) reinforce its clinical utility. Patients with higher LLEAP scores report better symptom outcome, supporting its relevance for capturing behaviors linked to therapeutic progress. These findings suggest that LLEAP not only aligns with engagement determinants and processes but also reflects outcomes that are meaningful for patients.

The final eight items of the LLEAP consistently emerged as top selections across 1,000 bootstrap samples and multiple cross-validation iterations, underscoring their stability and generalizability. Although the initial pool of 120 items spanned a range of engagement facets, from motivation and alliance to within-session behaviors (Table S8), the most frequently selected items were those reflecting between-session efforts (Table 1). This result suggests that, based solely on session transcripts, automated item selection tended toward prompts indicative of patients’ therapeutic work outside the session. Because the item selection process was primarily driven by correlations with all validation scales, it appears that these between-session indicators were especially predictive of motivation, alliance, and outcomes like symptom severity^45,46^. Intriguingly, the LLM did not have direct information about the patients’ actual behaviors outside the therapy room. It relied solely on session dialogue. Yet, what was said during the session, e.g., references to homework completion, follow-through on therapeutic strategies, or discussions of challenges and successes since the previous session, likely provided implicit verbal cues that corresponded to what patients were doing between sessions. Thus, the session content itself seems to encode meaningful signals about out-of-session effort and engagement, allowing the LLM-based approach to detect and make use of these indicators.

This study introduced a novel application of LLMs to psychometric scale development, offering an automated, scalable, and privacy-preserving approach to measuring psychological constructs in text data. However, as this is the first application, further testing is necessary. The strengths and limitations of this work can be discussed at different levels. The LLM rating scale approach relies on text input, overlooking non-verbal cues and unexpressed patient states, which are crucial in psychological therapies. Furthermore, adopting these automated methods requires computational resources and technical expertise, potentially restricting their use in some settings. Despite using locally run models to preserve privacy, the computational demands of larger models may challenge their accessibility. However, these limitations are offset by the approach’s ability to automate the rating process, reducing response burden and saving time and resources. Additionally, its grounding in psychological principles allows theory-driven prompt design that enhances face validity. Unlike machine learning methods that extract technical features (e.g., audio signals or video frames) that can capture subtle cues, but are hard to interpret^47^, LLM rating scales ensure interpretability by using conceptually clear items directly linked to the constructs of interest. The use of pre-trained, open-source models requiring only inference further simplifies implementation, making the system flexible and easily updatable with newer model versions. The reproducibility of the method is enhanced by its design, allowing prompts to be shared and used like traditional questionnaires. For example, the LLEAP items (Table 1 & Table S8) are freely available for other researchers, facilitating replication of the findings in diverse settings. The LLEAP scale also has limitations and its validation could be enhanced by comparisons with specialized engagement questionnaires such as the Treatment Engagement Rating (TER)^48^. Although we used session-level alliance measures from the BPSR for validation, future research could enhance construct validity by including more widely established alliance instruments such as the Working Alliance Inventory (WAI)^49^. Nonetheless, it demonstrates strong psychometric properties, including high internal consistency and acceptable model fit. Grounded in Holdsworth et al.’s Model of Client Engagement^18^, it aligns with constructs like motivation, alliance, and session efforts, enhancing its relevance for clinical research and practice.

At the study level, limitations include a relatively small patient-level sample compared to traditional validation studies and the significant computational demands associated with repeated validation procedures. However, this limitation is partially mitigated by the relatively large number of transcripts available, which enabled analyses of within-patient effects. The high number of session-level data provided insights into individual variability, enhancing the trustworthiness of the findings despite the smaller patient-level sample size. Automated transcription facilitated efficient and scalable data processing without compromising accuracy, as evidenced by the coherence of the results. The study employed rigorous techniques such as bootstrap optimism correction, cross-validation, and hierarchical modeling to ensure robust and generalizable findings^50^.

Among the patients, 12.9% had a personal or parental history of immigration, with birth countries including Algeria, China, France, Iran, Italy, Kazakhstan, Luxembourg, Poland, Russia, South Korea, Turkey, Ukraine, and the USA, while the remaining 87.1% were born in Germany, as were both of their parents. Data on ethnicity or race were not collected, as such questions are generally considered politically and historically inappropriate in Germany. The data comes from naturalistic routine outpatient care, which enhances ecological validity. While bootstrapping and cross-validation provided internal validation, external validation in more diverse populations and settings is essential to test the generalizability of the findings^51^. LLMs offer multilingual capabilities, allowing the LLM rating scale approach to be applied in different languages. Although primarily trained on English corpora, the model demonstrated effective transcription and rating in German. Future research should explore its validity in less common languages and linguistic contexts. LLEAP is freely available to support further validation efforts in different populations, languages, and research settings.

This study opens numerous avenues for future research. A comparative analysis of different LLMs would be particularly valuable, as it would also allow testing the stability of the LLM rating scale approach. While this study used the smaller Llama 3.1 8B model due to computational constraints, larger models such as the 70B version may yield improved accuracy and deeper insights. The DISCOVER framework allows researchers to easily choose between a variety of open-source LLMs. Fine-tuning LLMs on domain-specific datasets could further optimize their ability to generate contextually relevant and accurate ratings, tailoring the models to the unique needs of specific samples or clinical populations. Advancements in prompt generation and item selection offer additional potential. Automated item selection algorithms such as the R package *stuart*^52^, feature selection algorithms like ElasticNet^53^, or prompt selection frameworks such as DSPy^54^ for programming instead of prompting language models could streamline the refinement of prompts and items. Incorporating multimodal approaches represents another promising direction. Adding audio-visual data, such as vocal tone, facial expressions, or body language, to text-based ratings may enhance the richness and precision of engagement assessments^55^. These non-verbal cues, often critical in therapy, could provide complementary insights and improve the reliability of LLM-generated ratings. Contextual data is also a key consideration for improving LLM performance. Integrating metadata such as patient diagnoses, session goals, or previous therapeutic progress into the LLM’s input could achieve more accurate and relevant ratings.

Segment-level analyses present another opportunity to uncover moment-to-moment dynamics within therapy sessions. Technical refinements, such as exploring optimal token lengths for transcript segmentation, are also necessary. As computational capacities improve, providing greater context within segments may enhance the reliability and validity of LLM-generated ratings. Methods for confidence estimation, such as “not evaluable” flags or weighting less informative segments, could further improve measurement precision by focusing analyses on the most meaningful content. Finally, LLM-generated explanations and explainable AI (XAI) frameworks hold promise for improving the interpretability of LLM-based assessments^56^. While LLMs can generate explanations that offer valuable insights into constructs like engagement, these explanations are not causally linked to the ratings. Tools like Gemma Scope^57^ could help researchers better understand the internal processes of LLMs, bridging the gap between observed outputs and the mechanisms that produce them. Such advancements in interpretability will be crucial for fostering trust and transparency in the use of LLMs in clinical and research contexts.

This study highlights the potential of LLM-based ratings to support clinical practice across various therapy stages, provided patients consent to video analyses. One promising application lies in early treatment planning. LLEAP ratings can help guide treatment strategies during the initial phase of therapy by identifying engagement challenges. For instance, therapists could use these ratings to decide between adopting a motivation-oriented or problem-oriented strategy, tailoring their approach to the unique needs of individual patients^58^. Throughout therapy, ROM enhanced by LLM-based ratings could detect early warning signals of disengagement. Indicators such as low motivation or a poor alliance are critical signals of unexpected treatment responses, including stagnation or deterioration^22,59^. By identifying these risks early, therapists could make timely adjustments to their interventions. In addition, clinical support tools based on these ratings could provide actionable recommendations, such as motivational interviewing^60^ or motivational enhancement techniques^61^. Alternatively, when relational challenges arise, rupture-repair strategies could be suggested to strengthen the alliance^62^.

Beyond individual therapy sessions, LLM-based engagement ratings have the potential to enhance supervision and training. Supervisors could use these ratings to provide more targeted and data-informed feedback to therapists, focusing on critical areas such as improving the therapeutic alliance or fostering patient motivation. Integrating engagement ratings into broader feedback-informed therapy frameworks has the potential to enhance how therapists approach treatment. Continuous, data-informed insights into patient engagement allow for therapy that is adaptive and responsive to patient needs over time.

## Conclusion

This study introduced a novel approach to developing and evaluating rating scales based on ratings from LLMs instead of human responses (i.e., LLM rating scales). This automated measurement approach offers new possibilities for assessing latent constructs within text data, such as therapy session transcripts. In addition to establishing new standards for automated measurement, the study outlines a comprehensive pipeline for semi-automated transcription, item generation, and item selection that enhances scalability and efficiency. The utility of the LLM rating scale was demonstrated through the development and evaluation of a measure of patient engagement in psychological therapies (i.e., LLEAP). Results indicated that this approach can produce psychometrically robust scales with meaningful correlations to established and theoretically related questionnaires, supporting its validity. The flexibility of the framework allows it to adapt to newly released LLMs, suggesting that ongoing advances in foundational models may further enhance the efficacy of this approach. Importantly, this approach can be implemented locally, ensuring that confidential text data remains protected and private. This method adds a new, automated, and scalable tool to the psychological assessment toolkit, with the potential to be used to measure a wide range of psychological constructs within text data.

## Supplementary Information

Below is the link to the electronic supplementary material.

Supplementary Material 1

## Data Availability

The data on which study conclusions are based are not publicly available due to the sensitive nature of the information and the fact that participants did not provide explicit consent for data sharing. The Python code for the LLM rating scale pipeline as well as the R scripts for item selection and scale evaluation are available via https://doi.org/10.17605/OSF.IO/GD8YE. The DISCOVER framework and its NOVA graphical user interface are publicly available on GitHub and can be accessed at https://github.com/hcmlab/discover, https://github.com/hcmlab/discover-modules, and https://github.com/hcmlab/nova. The LLEAP items are freely available in Table 1 and Table S8.
