# Supplementary material for: Development and validation of large language model rating scales for automatically transcribed psychological therapy sessions
Source: Sci Rep. 2025 Aug 12;15:29541. doi: 10.1038/s41598-025-14923-y (PMC12343941; doi:10.1038/s41598-025-14923-y)
Supplement: Supplementary file 1 — Supplementary Material 1 [file 41598_2025_14923_MOESM1_ESM.pdf]

Supplemental Material

Supplementary Table S1

Diagnoses of Patients in the Original Sample

Diagnosis	Frequency (Percent)
Depressive Disorder/Dysthymia	83 (53.3%)
Panic/Agoraphobia	20 (13.0%)
Other Anxiety Disorder	14 (9.1%)
Adjustment Disorder	18 (11.7%)
Personality Disorder	8 (5.2%)
Other Disorder	12 (7.7%)
Total	155 (100%)

Multilevel Model Formulas and Descriptions

Session-Level Model

$$\text{Validation Scale}_{\text{Session Level}} = \gamma_{00} + \gamma_{10}(\text{LLEAP}_{\text{Patient Mean-Centered}}) + \gamma_{01}(\text{LLEAP}_{\text{Patient Mean}}) + u_{\text{Patient}} + \epsilon \quad \text{(S1)}$$

γ_{00}: Fixed intercept, representing expected validation scale score when $\text{LLEAP}_{\text{Patient Mean-Centered}} = 0$ and $\text{LLEAP}_{\text{Patient Mean}} = 0$.

γ_{10}: Effect of session-level deviations from the LLEAP patient mean on the validation scale.

γ_{01}: Effect of the patient mean LLEAP score across all sessions on the validation scale.

u_{Patient}: Random intercept accounting for variability in baseline levels across patients.

ϵ: Residual error term.

Cluster-robust standard errors were calculated using the *lmerTest* package to account for clustering within therapists (v3.1-3, Kuznetsova et al., 2017).

Patient-Level Model

$$\text{Validation Scale}_{\text{Patient Level}} = \gamma_{00} + \gamma_{10}(\text{LLEAP}_{\text{Patient Mean}}) + u_{\text{Therapist}} + \epsilon \quad \text{(S2)}$$

γ_{00}: Fixed intercept, representing expected validation scale score when $\text{LLEAP}_{\text{Patient Mean}} = 0$.

γ_{10}: Effect of the patient mean LLEAP score on the validation scale.

$u_{\text{Therapist}}$: Random intercept accounting for variability in baseline levels across therapists.

ϵ: Residual error term.

Supplementary Table S2

Descriptive Statistics of LLEAP and Its Validation Scales on the Patient Level in the Original Sample

Construct (Scale)	*Items*	*M*	*SD*	Median	Min	Max	Range	Skew	Kurtosis	K-S	α	ω	Missing
						LLM Rating Scale							
**Engagement (LLEAP)**	8	52.27	6.84	52.78	23.40	67.51	44.12	–0.73	1.35	0.06	.95	.95	0%
**Engagement Determinants:**						Concurrent Validation Scales							
• Motivation (ASC-MOT)	9	4.60	0.45	4.76	2.44	5.00	2.56	–2.02	4.86	0.19*	.83	.88	11.14%
• Alliance:													
◦ Patient-Rated Alliance (BPSR-P)	4	2.59	0.54	2.84	0.00	3.00	3.00	–1.71	3.23	0.22*	.86	.88	0.57%
◦ Therapist-Rated Alliance (BPSR-T)	3	2.12	0.55	2.22	0.17	2.97	2.80	–0.97	0.93	0.10	.87	.87	0.79%
**Engagement Processes:**													
• Within-Session Effort:													
◦ Problem Coping (BPSR-P)	3	0.85	1.09	0.92	–3.00	2.62	5.62	–0.93	1.13	0.08	.91	.91	0.57%
• Between-Session Effort:													
◦ Willingness (BPSR-T)	1	1.53	0.83	1.60	–1.25	3.00	4.25	–0.51	0.09	0.07	—	—	0.79%
**Engagement Outcome:**						Predictive Validation Scale							
• Symptom Outcome (OQ-30)	30	1.64	0.63	1.55	0.00	2.93	2.93	–0.07	–0.72	0.07	.95	.96	23.8%

*Note. N*p = 155 patients. The descriptives are based on the imputed data set. The Missing column indicates the percentage of missing values in the variables before imputation. LLEAP = Large Language Model Engagement Assessment in Psychological Therapies; ASC-MOT = Assessment for Signal Clients–Motivation subscale; BPSR-P = Patient-Rated Bern Post-Session Report; BPSR-T = Therapist-Rated Bern Post-Session Report; OQ-30 = Outcome Questionnaire-30. K-S = Kolmogorow-Smirnov test statistic. Asterisks indicate that the distribution of the variables deviates significantly from normality. α = Cronbach's alpha; ω = McDonald's omega. For LLEAP, α and ω were derived from multilevel confirmatory factor analysis in the original sample. Bootstrap optimism-corrected and cross-validated values can be found in Table 2. For the concurrent validation scales (Motivation, Alliance, Within- and Between-Session Effort), α and ω were calculated at session 15. For the predictive validation scale (Symptom Outcome), α and ω were calculated ten sessions later, after session 25.

Supplementary Table S3

Between-Patient Correlations of LLEAP and the Validation Scales in the Original Sample

Construct (Scale)	1	2	3	4	5	6	7
**Engagement (LLEAP)** [1]	—						
**Engagement Determinants:**							
• Motivation (ASC-MOT) [2]	.413 [.273, .536]	—					
• Alliance:							
◦ Patient-Rated Alliance (BPSR-P) [3]	.376 [.233, .504]	.606 [.496, .697]	—				
◦ Therapist-Rated Alliance (BPSR-T) [4]	.406 [.265, .530]	.332 [.184, .465]	.416 [.277, .539]	—			
**Engagement Processes:**							
• Within-Session Effort:							
◦ Problem Coping (BPSR-P) [5]	.337 [.190, .470]	.291 [.140, .429]	.406 [.265, .529]	.232 [.077, .376]	—		
• Between-Session Effort:							
◦ Willingness (BPSR-T) [6]	.390 [.248, .516]	.326 [.177, .460]	.387 [.245, .514]	.669 [.572, .748]	.323 [.174, .457]	—	
**Engagement Outcomes:**							
• Symptom Outcome (OQ-30) [7]	–.304 [–.440, –.154]	–.217 [–.362, –.062]	–.216 [–.361, –.060]	–.106 [–.260, .052]	–.473 [–.587, –.341]	–.211 [–.357, –.055]	—

Note. *N*p = 155. Correlation coefficients *r* and upper and lower limits of the 95% confidence intervals were optimism-corrected using a bootstrapping procedure with 1,000 bootstrap samples. LLEAP = Large Language Model Engagement Assessment in Psychological Therapies; ASC-MOT = Assessment for Signal Clients–Motivation subscale; BPSR-P = Patient-Rated Bern Post-Session Report; BPSR-T = Therapist-Rated Bern Post-Session Report; OQ-30 = Outcome Questionnaire-30.

Supplementary Table S4

Between-Patient Correlations of LLEAP and the Validation Scales with Bootstrap Optimism Correction

Construct (Scale)	1	2	3	4	5	6	7
**Engagement (LLEAP)** [1]	—						
**Engagement Determinants:**							
• Motivation (ASC-MOT) [2]	.381 [.258, .525]	—					
• Alliance:							
◦ Patient-Rated Alliance (BPSR-P) [3]	.347 [.217, .493]	.586 [.481, .686]	—				
◦ Therapist-Rated Alliance (BPSR-T) [4]	.387 [.250, .519]	.313 [.169, .455]	.398 [.262, .528]	—			
**Engagement Processes:**							
• Within-Session Effort:							
◦ Problem Coping (BPSR-P) [5]	.326 [.175, .459]	.279 [.125, .418]	.404 [.250, .519]	.222 [.062, .365]	—		
• Between-Session Effort:							
◦ Willingness (BPSR-T) [6]	.358 [.233, .505]	.302 [.162, .449]	.361 [.230, .503]	.644 [.557, .737]	.311 [.159, .446]	—	
**Engagement Outcomes:**							
• Symptom Outcome (OQ-30) [7]	–.283 [–.425, –.143]	–.216 [–.347, –.051]	–.209 [–.346, –.049]	–.099 [–.245, .041]	–.456 [–.572, –.330]	–.198 [–.342, –.045]	—

Note. *N*p = 155. Correlation coefficients *r* and upper and lower limits of the 95% confidence intervals were optimism-corrected using a bootstrapping procedure with 1,000 bootstrap samples. LLEAP = Large Language Model Engagement Assessment in Psychological Therapies; ASC-MOT = Assessment for Signal Clients–Motivation subscale; BPSR-P = Patient-Rated Bern Post-Session Report; BPSR-T = Therapist-Rated Bern Post-Session Report; OQ-30 = Outcome Questionnaire-30.

Supplementary Table S5

Between-Patient Correlations of LLEAP and the Validation Scales with Repeated 3-Fold Cross-Validation

Construct (Scale)	1	2	3	4	5	6	7
**Engagement (LLEAP)** [1]	—						
**Engagement Determinants:**							
• Motivation (ASC-MOT) [2]	.372<br>[.113, .584]	—					
• Alliance:							
◦ Patient-Rated Alliance (BPSR-P) [3]	.333<br>[.071, .554]	.585<br>[.376, .739]	—				
◦ Therapist-Rated Alliance (BPSR-T) [4]	.374<br>[.116, .586]	.319<br>[.054, .543]	.407<br>[.152, .611]	—			
**Engagement Processes:**							
• Within-Session Effort:							
◦ Problem Coping (BPSR-P) [5]	.304<br>[.035, .532]	.289<br>[.019, .519]	.410<br>[.155, .613]	.230<br>[-.042, .472]	—		
• Between-Session Effort:							
◦ Willingness (BPSR-T) [6]	.357<br>[.097, .572]	.312<br>[.045, .537]	.378<br>[.119, .590]	.669<br>[.485, .797]	.315<br>[.049, .541]	—	
**Engagement Outcomes:**							
• Symptom Outcome (OQ-30) [7]	– .276<br>[–.510, –.005]	–.218<br>[–.462, .056]	–.212<br>[–.459, .065]	–.101<br>[–.362, .175]	–.470<br>[–.657, –.227]	–.206<br>[–.453, .069]	—

Note. *N*p = 155 patients with ~52 patients per fold, with all patients contributing across folds. Correlation *r* and 95% confidence intervals were averaged across the repeated test fold. LLEAP = Large Language Model Engagement Assessment in Psychological Therapies; ASC-MOT = Assessment for Signal Clients–Motivation subscale; BPSR-P = Patient-Rated Bern Post-Session Report; BPSR-T = Therapist-Rated Bern Post-Session Report; OQ-30 = Outcome Questionnaire-30.

Supplementary Table S6

Correlations of LLEAP with the Validation Scales and Different Validation Methods

Construct (Scale)	Original Sample	Optimism Corrected	Cross Validated
**Engagement (LLEAP)**	—	—	—
**Engagement Determinants:**			
• Motivation (ASC-MOT)	.413<br>[.273, .536]	.381<br>[.258, .525]	.372<br>[.113, .584]
• Alliance:			
◦ Patient-Rated (BPSR-P)	.376<br>[.233, .504]	.347<br>[.217, .493]	.333<br>[.071, .554]
◦ Therapist-Rated (BPSR-T)	.406<br>[.265, .530]	.387<br>[.250, .519]	.374<br>[.116, .586]
**Engagement Processes:**			
• Within-Session Effort:			
◦ Problem Coping (BPSR-P)	.337<br>[.190, .470]	.326<br>[.175, .459]	.304<br>[.035, .532]
• Between-Session Effort:			
◦ Willingness (BPSR-T)	.390<br>[.248, .516]	.358<br>[.233, .505]	.357<br>[.097, .572]
**Engagement Outcomes:**			
• Symptom Outcome (OQ-30)	–.304<br>[–.440, –.154]	–.283<br>[–.425, –.143]	–.276<br>[–.510, –.005]

Note. Np = 155 patients. Correlations reflect the relationship between LLEAP scores and validation scales. LLEAP = Large Language Model Engagement Assessment in Psychological Therapies; ASC-MOT = Assessment for Signal Clients–Motivation subscale; BPSR-P = Patient-Rated Bern Post-Session Report; BPSR-T = Therapist-Rated Bern Post-Session Report; OQ-30 = Outcome Questionnaire-30. Original Sample: Estimates from the dataset without corrections. Optimism Corrected: Adjusted for overfitting via bootstrapping. Cross Validated: Derived from repeated three-fold cross-validation with 20 repeats to assess generalizability. Cross-validated values used approximately 52 patients per fold, with all patients contributing across folds. Values in brackets indicate 95% confidence intervals of the correlations.

Supplementary Table S7

Hierarchical Linear Model Results with LLEAP Predicting Validation Scales in Repeated 3-Fold Cross-Validation

Parameter	Mean Coefficient	Aggregated SE	Mean Lower 95% CI	Mean Upper 95% CI
		Engagement Determinants		
Motivation				
• Intercept	4.595*	0.061	4.475	4.717
• Patient mean	0.174*	0.058	0.059	0.289
Patient-Rated Alliance				
• Intercept	2.596*	0.069	2.461	2.731
• Patient mean-centered	0.041*	0.017	0.008	0.073
• Patient mean	0.167*	0.087	0.007	0.326
Therapist-Rated Alliance				
• Intercept	2.133*	0.070	1.996	2.270
• Patient mean-centered	0.052*	0.024	0.005	0.098
• Patient mean	0.193*	0.079	0.042	0.344
		Engagement Processes		
Within-Session Effort:				
• Problem Coping				
◦ Intercept	0.862*	0.145	0.579	1.145
◦ Patient mean-centered	0.121*	0.044	0.036	0.205
◦ Patient mean	0.305*	0.129	0.055	0.555
Between-Session Effort:				
• Willingness				
◦ Intercept	1.553*	0.105	1.347	1.760
◦ Patient mean-centered	0.094*	0.048	0.002	0.187
◦ Patient mean	0.278*	0.110	0.065	0.490
		Engagement Outcomes		
Symptom Outcome				
• Intercept	1.652*	0.089	1.475	1.830
• Patient mean	–0.177*	0.084	–0.344	–0.009

Note. Np = 155. Cross-validated values used ~52 patients per fold, with all patients contributing across folds. Three-fold cross-validation was used with 20 repeats. LLEAP = Large Language Model Engagement Assessment in Psychological Therapies. HLM results were aggregated by calculating the mean of coefficients, confidence interval bounds, and the standard error derived from the root mean square of individual SEs across groups. For session-level validation scales (Alliance, Within-, & Between-Session Effort), models included patient mean-centered LLEAP as a Level 1 predictor and patient mean LLEAP as a Level 2 predictor, accounting for clustering within patients and using cluster-robust standard errors for therapist nesting. For patient-level variables (motivation, symptom outcomes), models used patient mean LLEAP as a predictor, with clustering accounted for at the therapist level. Effects marked with * indicate that the mean 95% confidence interval does not include zero, suggesting statistical significance at the .05 level.

Supplementary Table S8

LLEAP 120-item pool

No	Facet	Variable Name	Prompt
		Patient Motivation (mot)	
1	Absorbed	eng_det_mot_abs	Please rate how absorbed the patient is by the therapy process on a scale from 0 (not absorbed at all) to 100 (very much absorbed)
2	Aligned	eng_det_mot_ali	Please rate how aligned the patient feels with the therapy goals on a scale from 0 (not aligned at all) to 100 (perfectly aligned)
3	Ardent	eng_det_mot_ard	Please rate how ardent the patient is about their therapeutic progress on a scale from 0 (not ardent at all) to 100 (extremely ardent)
4	Committed	eng_det_mot_com	Please rate how committed the patient is to the therapy process on a scale from 0 (not committed at all) to 100 (fully committed)
5	Commitment to Goals	eng_det_mot_ctg	Please rate how committed the patient is to achieving their therapy goals on a scale from 0 (not committed at all) to 100 (fully committed)
6	Curious	eng_det_mot_cur	Please rate how curious the patient is about exploring new therapeutic ideas on a scale from 0 (not curious at all) to 100 (very curious)
7	Dedicated	eng_det_mot_ded	Please rate how dedicated the patient is to achieving their therapy goals on a scale from 0 (not dedicated at all) to 100 (fully dedicated)
8	Determined	eng_det_mot_det	Please rate how determined the patient is to overcome their challenges on a scale from 0 (not determined at all) to 100 (very determined)
9	Driven	eng_det_mot_dri	Please rate how driven the patient is towards personal growth through therapy on a scale from 0 (not driven at all) to 100 (very driven)
10	Eager	eng_det_mot_eag	Please rate how eager the patient is to engage in therapy sessions on a scale from 0 (not eager at all) to 100 (extremely eager)
11	Enthusiastic	eng_det_mot_ent	Please rate how enthusiastic the patient is about continuing therapy on a scale from 0 (not enthusiastic at all) to 100 (very enthusiastic)
12	Inspired	eng_det_mot_ins	Please rate how inspired the patient is by the therapy sessions on a scale from 0 (not inspired at all) to 100 (greatly inspired)
13	Keen	eng_det_mot_kee	Please rate how keen the patient is to progress in therapy on a scale from 0 (not keen at all) to 100 (extremely keen)
14	Motivated	eng_det_mot_mot	Please rate how motivated the patient is to engage in therapy on a scale from 0 (not motivated at all) to 100 (highly motivated)

No	Facet	Variable Name	Prompt
15	Passionate	eng_det_mot_pas	Please rate how passionate the patient is about their therapeutic journey on a scale from 0 (not passionate at all) to 100 (very passionate)
16	Persistent	eng_det_mot_per	Please rate how persistent the patient is in overcoming therapeutic challenges on a scale from 0 (not persistent at all) to 100 (extremely persistent)
17	Purposeful	eng_det_mot_pur	Please rate how purposeful the patient is in pursuing therapy goals on a scale from 0 (not purposeful at all) to 100 (highly purposeful)
18	Single-minded	eng_det_mot_sin	Please rate how single-minded the patient is in focusing on therapy outcomes on a scale from 0 (easily distracted) to 100 (completely focused)
19	Tenacious	eng_det_mot_ten	Please rate how tenacious the patient is in pursuing therapeutic goals despite difficulties on a scale from 0 (not tenacious at all) to 100 (extremely tenacious)
20	Willing	eng_det_mot_wil	Please rate how willing the patient is to engage in therapy on a scale from 0 (not willing at all) to 100 (highly willing to engage)
21	Zealous	eng_det_mot_zea	Please rate how zealous the patient is about applying therapeutic techniques on a scale from 0 (not zealous at all) to 100 (extremely zealous)
		Therapeutic Relationship (rel)	
22	Bonded	eng_det_rel_bon	Please rate how bonded the patient feels with the therapist on a scale from 0 (not bonded at all) to 100 (strongly bonded)
23	Collaboration	eng_det_rel_cl1	Please rate the level of collaboration between the patient and the therapist in setting and working towards goals on a scale from 0 (no collaboration) to 100 (high collaboration)
24	Collaboration	eng_det_rel_cl2	Please rate how collaborative the patient is in the therapeutic process on a scale from 0 (not collaborative at all) to 100 (highly collaborative)
25	Collaboration	eng_det_rel_cl3	Please rate how often the patient resists advice from the therapist on a scale from 0 (always resists) to 100 (never resists)
26	Collaboration	eng_det_rel_cl4	Please rate how actively the patient participates in managing their illness on a scale from 0 (not active at all) to 100 (very active)
27	Communicative	eng_det_rel_cmm	Please rate how communicative the patient is during therapy sessions on a scale from 0 (not communicative at all) to 100 (very communicative)
28	Connected	eng_det_rel_con	Please rate how connected the patient feels to the therapist on a scale from 0 (not connected at all) to 100 (very connected)
29	Emotional Bond	eng_det_rel_emo	Please rate the strength of the emotional bond between the patient and the therapist on a scale from 0 (no bond) to 100 (very strong bond)

No	Facet	Variable Name	Prompt
30	Empathy	eng_det_rel_emp	Please rate how empathetic the patient perceives the therapist on a scale from 0 (not empathetic) to 100 (highly empathetic)
31	Harmonious	eng_det_rel_har	Please rate how harmonious the relationship between the patient and therapist is on a scale from 0 (not harmonious at all) to 100 (perfectly harmonious)
32	In-tune	eng_det_rel_itu	Please rate how in-tune the patient is with the therapeutic goals on a scale from 0 (not in-tune at all) to 100 (perfectly in-tune)
33	Openness to Feedback	eng_det_rel_otf	Please rate how open the patient is to receiving and discussing feedback from the therapist on a scale from 0 (not open at all) to 100 (completely open)
34	Rapport Quality	eng_det_rel_rap	Please rate the overall quality of the therapeutic rapport between the patient and the therapist on a scale from 0 (poor rapport) to 100 (excellent rapport)
35	Respect	eng_det_rel_res	Please rate the extent to which the patient feels respected by the therapist on a scale from 0 (no respect) to 100 (full respect)
36	Safety	eng_det_rel_saf	Please rate to what extent the patient feels safe and supported in the therapy sessions on a scale from 0 (not safe at all) to 100 (completely safe)
37	Therapeutic Alliance	eng_det_rel_tha	Please rate how strong the therapeutic alliance is between the patient and the therapist on a scale from 0 (weak alliance) to 100 (strong alliance)
38	Trust	eng_det_rel_trs	Please rate how much the patient trusts the therapist to share their innermost thoughts on a scale from 0 (no trust) to 100 (complete trust)
39	Trust	eng_det_rel_tru	Please rate the level of trust the patient has in the therapist on a scale from 0 (no trust) to 100 (complete trust)
		Patients' Minimum Effort: Attendance (eff)	
40	Attendance	eng_pro_eff_att	Please rate how consistent the patient is in attending therapy sessions on a scale from 0 (rarely attends) to 100 (always attends)
41	Availability	eng_pro_eff_ava	Please rate how easy it is to arrange appointments with the patient on a scale from 0 (very difficult) to 100 (very easy)
42	Availability	eng_pro_eff_avy	Please rate how likely the patient is to avoid making appointments on a scale from 0 (always avoids) to 100 (never avoids)
43	Punctuality	eng_pro_eff_pun	Please rate how punctual the patient is for therapy sessions on a scale from 0 (never on time) to 100 (always on time)
44	Session Attendance	eng_pro_eff_sat	Please rate how consistently the patient attends scheduled therapy sessions on a scale from 0 (never attends) to 100 (always attends)
45	Timeliness	eng_pro_eff_tim	Please rate how punctual the patient is for therapy sessions on a scale from 0 (always late) to 100 (always on time)

No	Facet	Variable Name	Prompt
Patients' Observable, Voluntary, Active Efforts Within Sessions: Participation or Involvement (wis)			
46	Active	eng_pro_wis_act	Please rate how active the patient is in therapy on a scale from 0 (not active at all) to 100 (very active)
47	Adaptability	eng_pro_wis_ada	Please rate how adaptable the patient is to changes in the therapeutic approach or plan on a scale from 0 (not adaptable at all) to 100 (very adaptable)
48	Attentive	eng_pro_wis_atv	Please rate how attentive the patient is during therapy sessions on a scale from 0 (not attentive at all) to 100 (highly attentive)
49	Behavioral Changes	eng_pro_wis_bec	Please rate how well the patient implements behavioral changes discussed in therapy on a scale from 0 (not at all) to 100 (very well)
50	Concentrated	eng_pro_wis_cnc	Please rate how concentrated the patient is during therapy sessions on a scale from 0 (not concentrated at all) to 100 (highly concentrated)
51	Communication of Fears	eng_pro_wis_cof	Please rate how openly the patient communicates their fears and anxieties during therapy sessions on a scale from 0 (not open at all) to 100 (completely open)
52	Contributing	eng_pro_wis_ctr	Please rate how much the patient contributes to the therapy sessions on a scale from 0 (not contributing at all) to 100 (actively contributing)
53	Comfort with Emotions	eng_pro_wis_cwe	Please rate how comfortable the patient is in expressing difficult emotions during therapy on a scale from 0 (not comfortable at all) to 100 (very comfortable)
54	Depth of Emotional Expression	eng_pro_wis_dee	Please rate how deeply the patient expresses their emotions during therapy sessions on a scale from 0 (not deeply at all) to 100 (very deeply)
55	Depth of Sharing	eng_pro_wis_dep	Please rate how deeply the patient delves into personal issues during therapy on a scale from 0 (not at all) to 100 (very deeply)
56	Disclosure	eng_pro_wis_dis	Please rate how willing the patient is to disclose personal information during therapy on a scale from 0 (not willing at all) to 100 (very willing)
57	Depth of Conversations	eng_pro_wis_doc	Please rate how often the patient engages in deep, meaningful conversations during therapy on a scale from 0 (never) to 100 (always)
58	Effort in Therapy	eng_pro_wis_eit	Please rate the amount of effort the patient puts into therapy sessions on a scale from 0 (no effort) to 100 (maximum effort)
59	Emotional Sharing	eng_pro_wis_ems	Please rate how openly the patient shares their emotions during therapy sessions on a scale from 0 (not open at all) to 100 (completely open)
60	Entranced	eng_pro_wis_enc	Please rate how entranced the patient is during deeply emotional sessions on a scale from 0 (not entranced at all) to 100 (completely entranced)

No	Facet	Variable Name	Prompt
61	Engaged	eng_pro_wis_eng	Please rate how engaged the patient is in the therapy process on a scale from 0 (not engaged at all) to 100 (fully engaged)
62	Engrossed	eng_pro_wis_ens	Please rate how engrossed the patient is in the therapy topics on a scale from 0 (not engrossed at all) to 100 (deeply engrossed)
63	Engagement with Self-care	eng_pro_wis_ews	Please rate how actively the patient engages with self-care practices recommended during therapy on a scale from 0 (never engages) to 100 (always engages)
64	Expressiveness	eng_pro_wis_exp	Please rate how expressive the patient is about their inner experiences during therapy sessions on a scale from 0 (not expressive at all) to 100 (very expressive)
65	Focused	eng_pro_wis_foc	Please rate how focused the patient is during therapy discussions on a scale from 0 (not focused at all) to 100 (extremely focused)
66	Honesty	eng_pro_wis_hon	Please rate how honest the patient is when discussing their feelings and thoughts during therapy on a scale from 0 (not honest at all) to 100 (completely honest)
67	Help Seeking	eng_pro_wis_hs1	Please rate how often the patient seeks help when needed on a scale from 0 (never) to 100 (always)
68	Help Seeking	eng_pro_wis_hs2	Please rate how difficult the patient finds it to ask for help on a scale from 0 (very difficult) to 100 (very easy)
69	Help Seeking	eng_pro_wis_hs3	Please rate how often the patient seeks help to prevent a crisis on a scale from 0 (never) to 100 (always)
70	Help Seeking	eng_pro_wis_hs4	Please rate how actively the patient seeks help on a scale from 0 (not active at all) to 100 (very active)
71	Honest Self-Assessment	eng_pro_wis_hsa	Please rate how honestly the patient assesses their own actions and behaviors during therapy on a scale from 0 (not honest at all) to 100 (completely honest)
72	Immersed	eng_pro_wis_imm	Please rate how immersed the patient is in understanding their behaviors during therapy on a scale from 0 (not immersed at all) to 100 (fully immersed)
73	Interacting	eng_pro_wis_int	Please rate how often the patient interacts with the therapist during sessions on a scale from 0 (rarely interacts) to 100 (frequently interacts)
74	Involved	eng_pro_wis_inv	Please rate how involved the patient is in the therapy process on a scale from 0 (not involved at all) to 100 (deeply involved)
75	Implementation of Feedback	eng_pro_wis_iof	Please rate how well the patient implements feedback from the therapist on a scale from 0 (does not implement) to 100 (fully implements)
76	Interactive	eng_pro_wis_itv	Please rate how interactive the patient is in the therapeutic process on a scale from 0 (not interactive at all) to 100 (highly interactive)
77	Openness to Discuss Problems	eng_pro_wis_odp	Please rate how open the patient is to discussing their personal problems during therapy on a scale from 0 (not open at all) to 100 (very open)

No	Facet	Variable Name	Prompt
78	Participating	eng_pro_wis_par	Please rate how actively the patient is participating in therapy sessions on a scale from 0 (not participating at all) to 100 (actively participating)
79	Perceptive	eng_pro_wis_pec	Please rate how perceptive the patient is to insights gained during therapy on a scale from 0 (not perceptive at all) to 100 (highly perceptive)
80	Proactive	eng_pro_wis_pr1	Please rate how proactive the patient is in addressing issues during therapy on a scale from 0 (not proactive at all) to 100 (highly proactive)
81	Present	eng_pro_wis_pre	Please rate how present the patient is during therapy sessions on a scale from 0 (not present at all) to 100 (fully present)
82	Reflectiveness	eng_pro_wis_rfl	Please rate how reflective the patient is about their own behaviors and thoughts during therapy sessions on a scale from 0 (not reflective at all) to 100 (highly reflective)
83	Responsive	eng_pro_wis_rsv	Please rate how responsive the patient is to feedback during therapy on a scale from 0 (not responsive at all) to 100 (highly responsive)
84	Studious	eng_pro_wis_stu	Please rate how studious the patient is in learning from therapy sessions on a scale from 0 (not studious at all) to 100 (extremely studious)
85	Supportive	eng_pro_wis_sup	Please rate how supportive the patient is in fostering a positive therapy environment on a scale from 0 (not supportive at all) to 100 (very supportive)
86	Vulnerability	eng_pro_wis_vul	Please rate how vulnerable the patient allows themselves to be during therapy sessions on a scale from 0 (not vulnerable at all) to 100 (completely vulnerable)
	Patients' Voluntary, Active Efforts Between Sessions: Homework or Practice (bts)		
87	Accountable	eng_pro_bts_acc	Please rate how accountable the patient is for their homework and practice activities on a scale from 0 (not accountable at all) to 100 (fully accountable)
88	Applying Techniques	eng_pro_bts_app	Please rate how often the patient applies therapeutic techniques outside of sessions on a scale from 0 (never) to 100 (always)
89	Assiduous	eng_pro_bts_ass	Please rate how assiduous the patient is in following therapeutic advice on a scale from 0 (not assiduous at all) to 100 (very assiduous)
90	Conscientious	eng_pro_bts_cns	Please rate how conscientious the patient is about applying therapeutic techniques outside of sessions on a scale from 0 (not conscientious at all) to 100 (highly conscientious)
91	Diligent	eng_pro_bts_dil	Please rate how diligent the patient is in completing therapy homework on a scale from 0 (not diligent at all) to 100 (extremely diligent)
92	Effort Between Sessions	eng_pro_bts_eff	Please rate how much effort the patient puts into therapeutic activities between sessions on a scale from 0 (no effort) to 100 (maximum effort)

No	Facet	Variable Name	Prompt
93	Embraced	eng_pro_bts_emb	Please rate how much the patient has embraced the therapeutic methods suggested on a scale from 0 (not embraced at all) to 100 (fully embraced)
94	Energized	eng_pro_bts_ene	Please rate how energized the patient feels after therapy sessions on a scale from 0 (not energized at all) to 100 (very energized)
95	Engaged in Homework	eng_pro_bts_enw	Please rate how engaged the patient is in completing therapy homework on a scale from 0 (not engaged at all) to 100 (fully engaged)
96	Empowered	eng_pro_bts_epw	Please rate how empowered the patient feels by the therapy on a scale from 0 (not empowered at all) to 100 (fully empowered)
97	Engagement with Techniques	eng_pro_bts_ewt	Please rate how actively the patient engages with therapeutic techniques outside of sessions on a scale from 0 (not engaged at all) to 100 (fully engaged)
98	Follow-through	eng_pro_bts_fth	Please rate how well the patient follows through with therapeutic recommendations outside of sessions on a scale from 0 (never follows through) to 100 (always follows through)
99	Homework Completion	eng_pro_bts_hwc	Please rate how consistent the patient is in completing assigned therapy homework on a scale from 0 (never completes) to 100 (always completes)
100	Implementation	eng_pro_bts_imp	Please rate how well the patient implements the strategies discussed in therapy on a scale from 0 (not at all) to 100 (very well)
101	Influenced	eng_pro_bts_inf	Please rate how influenced the patient is by the therapist's guidance on a scale from 0 (not influenced at all) to 100 (highly influenced)
102	Initiating	eng_pro_bts_ini	Please rate how often the patient initiates topics or discussions in therapy on a scale from 0 (never initiates) to 100 (always initiates)
103	Initiative	eng_pro_bts_ive	Please rate how much initiative the patient shows in applying what they learn in therapy to their daily life on a scale from 0 (no initiative) to 100 (high initiative)
104	Motivated for Homework	eng_pro_bts_mhw	Please rate how motivated the patient is to complete their therapy homework on a scale from 0 (not motivated at all) to 100 (highly motivated)
105	Mindful	eng_pro_bts_min	Please rate how mindful the patient is of their thoughts and feelings during therapy on a scale from 0 (not mindful at all) to 100 (very mindful)
106	Observant	eng_pro_bts_obs	Please rate how observant the patient is of changes in their mood or behavior as a result of therapy on a scale from 0 (not observant at all) to 100 (very observant)
107	Occupied	eng_pro_bts_occ	Please rate how occupied the patient is with applying therapeutic techniques in daily life on a scale from 0 (not occupied at all) to 100 (fully occupied)

No	Facet	Variable Name	Prompt
108	Onboard	eng_pro_bts_onb	Please rate how onboard the patient is with the therapist's recommendations on a scale from 0 (not onboard at all) to 100 (fully onboard)
109	Participation in Exercises	eng_pro_bts_pie	Please rate how often the patient participates in therapeutic exercises or activities assigned during sessions on a scale from 0 (never participates) to 100 (always participates)
110	Plugged-in	eng_pro_bts_plu	Please rate how plugged-in the patient is to following up on therapy sessions on a scale from 0 (not plugged-in at all) to 100 (completely plugged-in)
111	Proactive	eng_pro_bts_pr2	Please rate how proactive the patient is in following up on therapy sessions on a scale from 0 (not proactive at all) to 100 (highly proactive)
112	Proactive Communication	eng_pro_bts_prc	Please rate how proactive the patient is in communicating progress or challenges between sessions on a scale from 0 (never communicates) to 100 (always communicates)
113	Ready	eng_pro_bts_rea	Please rate how ready the patient is to engage in each therapy session on a scale from 0 (not ready at all) to 100 (completely ready)
114	Receptive	eng_pro_bts_rec	Please rate how receptive the patient is to new therapy approaches on a scale from 0 (not receptive at all) to 100 (very receptive)
115	Reflective	eng_pro_bts_ref	Please rate how often the patient reflects on therapy sessions outside of scheduled times on a scale from 0 (never) to 100 (always)
116	Reflection on Sessions	eng_pro_bts_ros	Please rate how often the patient reflects on the content of therapy sessions outside of scheduled times on a scale from 0 (never reflects) to 100 (always reflects)
117	Session Preparation	eng_pro_bts_spr	Please rate how prepared the patient is for therapy sessions on a scale from 0 (not prepared at all) to 100 (fully prepared)
118	Synchronized	eng_pro_bts_syn	Please rate how synchronized the patient is with the therapist's timing and pacing during therapy on a scale from 0 (not synchronized at all) to 100 (perfectly synchronized)
119	Vested	eng_pro_bts_ves	Please rate how vested the patient is in the outcomes of their therapy on a scale from 0 (not vested at all) to 100 (deeply vested)
120	Vigilant	eng_pro_bts_vig	Please rate how vigilant the patient is in maintaining therapeutic gains on a scale from 0 (not vigilant at all) to 100 (very vigilant)

Note. Each prompt concluded with the following instruction: 'Please respond in JSON format: {"rating": [insert rating between 0-100 here]}. Do not write anything else.' LLEAP = Large Language Model Engagement Assessment in Psychological Therapies.

Item Generation: Chat History With ChatGPT 4o

Hi ChatGPT, I am a psychotherapy researcher and I am interested in client or patient engagement and disengagement in psychotherapy. Specifically, I would like to measure or evaluate client engagement and disengagement by asking large language models about it based on psychotherapy transcripts of patient statements in therapy sessions. To create an item pool of prompts about clients' engagement and disengagement, I would first like to think about the facets, components and definitions that constitute engagement and disengagement. I know that the term engagement is sometimes used differently in psychotherapy research. Which definitions of engagement (or disengagement) do you know? Moreover, what are the most important aspects, facets or components of engagement and disengagement?

Engagement and disengagement in psychotherapy are multifaceted constructs that can be understood and measured in various ways. Here are some definitions and key aspects of these constructs:

Definitions of Engagement

1. **Behavioral Engagement**:
    - Involves the client's active participation in therapy sessions, adherence to homework assignments, and consistency in attending sessions.
2. **Emotional Engagement**:
    - Refers to the client's emotional involvement and investment in the therapeutic process, including expressions of feelings and emotional connection with the therapist.
3. **Cognitive Engagement**:
    - Involves the client's active cognitive participation, such as reflecting on issues discussed in therapy, demonstrating insight, and applying therapeutic concepts outside sessions.
4. **Relational Engagement**:
    - The quality of the therapeutic alliance between the client and therapist, encompassing trust, rapport, and mutual respect.
5. **Motivational Engagement**:
    - The client's motivation to change, their readiness to engage in therapeutic work, and their commitment to the therapeutic goals.

Definitions of Disengagement

1. **Behavioral Disengagement**:
    - Characterized by missed sessions, lack of participation in therapeutic activities, and non-adherence to treatment plans.
2. **Emotional Disengagement**:

  - The client's emotional detachment or withdrawal from the therapeutic process, reluctance to share feelings, or a lack of emotional response.
3. **Cognitive Disengagement**:
   - The client's lack of cognitive involvement, such as resistance to exploring thoughts and beliefs, or not applying therapeutic insights.
4. **Relational Disengagement**:
   - Poor therapeutic alliance, marked by lack of trust, rapport, or collaboration with the therapist.
5. **Motivational Disengagement**:
   - Low motivation to engage in the therapeutic process, lack of commitment to change, and minimal effort in pursuing therapeutic goals.

Key Aspects, Facets, and Components of Engagement and Disengagement

Engagement

1. **Consistency and Regularity**:
   - Frequency and punctuality in attending sessions.
2. **Active Participation**:
   - Involvement in discussions, engagement in therapeutic exercises, and completing homework.
3. **Openness and Honesty**:
   - Willingness to share personal thoughts, feelings, and experiences.
4. **Therapeutic Alliance**:
   - Building a strong, collaborative relationship with the therapist.
5. **Motivation and Goal Orientation**:
   - Setting and pursuing therapy goals, showing a desire for personal growth and change.
6. **Responsiveness to Therapy**:
   - Showing signs of progress, responding to therapeutic interventions, and reflecting on therapy content.
7. **Cognitive Involvement**:
   - Engaging in self-reflection, demonstrating insight, and applying therapy concepts in daily life.
8. **Emotional Expression**:
   - Expressing and processing emotions during sessions.

Disengagement

1. **Session Attendance Issues**:
   - Frequent cancellations, no-shows, or inconsistent attendance.
2. **Passive or Minimal Participation**:

    - Limited involvement in discussions, reluctance to engage in therapeutic activities, and incomplete homework.
3. **Emotional Withdrawal**:
    - Reluctance to express emotions, minimal emotional response, or emotional detachment.
4. **Poor Therapeutic Alliance**:
    - Lack of trust, rapport, or cooperation with the therapist.
5. **Resistance to Change**:
    - Defensiveness, reluctance to set or pursue goals, and minimal effort in therapy.
6. **Lack of Insight or Reflection**:
    - Difficulty in self-reflection, resistance to exploring thoughts and beliefs, and not applying therapeutic insights.
7. **Externalizing Blame**:
    - Attributing problems to external factors rather than taking personal responsibility.
8. **Minimal Emotional Involvement**:
    - Lack of emotional engagement or investment in the therapeutic process.

Evaluating Engagement and Disengagement

To evaluate client engagement and disengagement using large language models based on psychotherapy transcripts, you could develop a set of prompts targeting these facets and components. Examples of such prompts might include:

1. **Behavioral Engagement**:
    - "How frequently does the client attend therapy sessions?"
    - "Does the client complete assigned homework or therapeutic tasks?"
2. **Emotional Engagement**:
    - "How openly does the client express their emotions during sessions?"
    - "Does the client appear emotionally invested in the therapy process?"
3. **Cognitive Engagement**:
    - "How reflective is the client about their thoughts and behaviors discussed in therapy?"
    - "Does the client apply therapeutic insights to their daily life?"
4. **Relational Engagement**:
    - "How would you describe the level of trust and rapport between the client and therapist?"
    - "Does the client collaborate with the therapist in setting and pursuing goals?"
5. **Motivational Engagement**:
    - "How committed is the client to making changes through therapy?"
    - "Does the client demonstrate a desire for personal growth?"

By considering these definitions and components, you can develop a comprehensive item pool to measure and evaluate client engagement and disengagement in psychotherapy.

Great, very nice, thank you very much! That seems to me to be a very reasonable definition and to cover many important facets of engagement and disengagement. I have also become aware of the following article on the topic of engagement. What elements of engagement are identified in them and how is engagement operationalized in them?

Holdsworth, E., Bowen, E., Brown, S. and Howat, D (2014). Client engagement in psychotherapeutic treatment and associations with client characteristics, therapist characteristics, and treatment factors. https://doi.org/10.1016/j.cpr.2014.06.004

The model of client engagement in psychotherapy, as presented in the article by Holdsworth et al. (2014), provides a comprehensive framework for understanding the factors influencing engagement, the process of engagement itself, and the outcomes associated with engagement. Here is a summary of the key components of the model:

Engagement Determinant Variables

1. **Clients' Motivation**: The client's intrinsic and extrinsic motivations to engage in therapy. This includes their readiness for change and personal goals.
2. **Therapeutic Relationship**: The quality of the relationship between the therapist and the client, characterized by trust, empathy, and collaboration. A strong therapeutic alliance is crucial for client engagement.

Engagement Process Variables

1. **Clients' Minimum Effort: Attendance**: Regular attendance at therapy sessions is the foundational level of engagement. Consistent attendance indicates a basic level of commitment to the therapeutic process.
2. **Clients' Observable, Voluntary, Active Efforts Within Sessions: Participation or Involvement**: This dimension captures the client's active engagement during sessions, such as participating in discussions, expressing emotions, and working collaboratively with the therapist.
3. **Clients' Voluntary, Active Efforts Between Sessions: Homework or Practice**: Engagement also includes the client's efforts outside of therapy sessions. This involves completing homework assignments, practicing new skills, and applying therapeutic insights in their daily life.

Engagement Outcome Variables

1. **Treatment Satisfaction**: The client's satisfaction with the therapy process and their perception of the therapeutic relationship and interventions.

2. **Amounts/Degrees of Changes in Functioning (Treatment Outcomes)**: This includes measurable changes in the client's psychological, emotional, and behavioral functioning as a result of the therapy. Positive outcomes indicate effective engagement and successful therapeutic interventions.

Summary of the Model

The model suggests that engagement in psychotherapy is influenced by the client's motivation and the quality of the therapeutic relationship. Engagement is operationalized through consistent attendance, active participation in sessions, and proactive efforts between sessions. The ultimate outcomes of engagement are client satisfaction with treatment and meaningful changes in functioning.

This model emphasizes the dynamic and multifaceted nature of engagement in psychotherapy, highlighting the interplay between client and therapist factors, the engagement process, and the resulting therapeutic outcomes.

Excellent, thank you! I would like to start developing or designing prompts for the large language models with you. Please remember, I want to assess the different facets of engagement (and disengagement) by asking the LLM on the basis of patients' and therapists' statements in psychotherapy session transcripts. I would like to focus on Engagement Determinant Variables first. I would like to design the prompts like this for example:

Please rate how motivated the patient is to engage in therapy on a scale from 0 (not motivated to engage at all) to 100 (highly motivated to engage).

Can you please start by recommending 10 such prompts for the two Engagement Determinant Variables (1. Clients' motivation and 2. Therapeutic Relationship), respectively.

Certainly! Here are ten prompts for each of the two Engagement Determinant Variables: Clients' motivation and Therapeutic Relationship.

Engagement Determinant Variable 1: Clients' Motivation

1. **Motivation Scale**: "Please rate how motivated the patient is to engage in therapy on a scale from 0 (not motivated to engage at all) to 100 (highly motivated to engage)."
2. **Motivation Commitment**: "How committed does the patient appear to be towards the therapeutic process? Rate from 0 (not committed at all) to 100 (extremely committed)."
3. **Change Willingness**: "Assess the patient's willingness to change their current behavior or thought patterns. Rate from 0 (completely unwilling) to 100 (fully willing)."
4. **Goal Orientation**: "Evaluate the clarity and specificity of the patient's therapeutic goals. Rate from 0 (no clear goals) to 100 (very clear and specific goals)."

5. **Effort Investment**: "How much effort does the patient put into therapy sessions? Rate from 0 (no effort) to 100 (maximum effort)."
6. **Personal Responsibility**: "To what extent does the patient take personal responsibility for their progress in therapy? Rate from 0 (not at all) to 100 (completely)."
7. **Initiative**: "Assess the patient's initiative in bringing up topics or issues to discuss in therapy. Rate from 0 (no initiative) to 100 (high initiative)."
8. **Openness to Feedback**: "How open is the patient to receiving and acting upon feedback from the therapist? Rate from 0 (completely closed off) to 100 (very open)."
9. **Self-Reflection**: "Evaluate the patient's ability to self-reflect and gain insights from therapy sessions. Rate from 0 (no self-reflection) to 100 (high level of self-reflection)."
10. **Motivational Statements**: "Based on the patient's statements, how often do they express motivation to improve or change? Rate from 0 (never) to 100 (always)."

Engagement Determinant Variable 2: Therapeutic Relationship

1. **Trust Level**: "Please rate the level of trust the patient has in the therapist on a scale from 0 (no trust) to 100 (complete trust)."
2. **Rapport Quality**: "How would you rate the overall quality of the therapeutic rapport between the patient and the therapist? Rate from 0 (poor rapport) to 100 (excellent rapport)."
3. **Empathy Perception**: "Assess how empathetic the patient perceives the therapist to be. Rate from 0 (not empathetic) to 100 (highly empathetic)."
4. **Communication Openness**: "Evaluate how openly the patient communicates with the therapist. Rate from 0 (very closed) to 100 (very open)."
5. **Therapeutic Alliance**: "How strong is the therapeutic alliance between the patient and the therapist? Rate from 0 (weak alliance) to 100 (strong alliance)."
6. **Safety Feeling**: "To what extent does the patient feel safe and supported in the therapy sessions? Rate from 0 (not safe at all) to 100 (completely safe)."
7. **Collaboration Level**: "Assess the level of collaboration between the patient and the therapist in setting and working towards goals. Rate from 0 (no collaboration) to 100 (high collaboration)."
8. **Therapist Understanding**: "How well does the patient feel understood by the therapist? Rate from 0 (not understood at all) to 100 (completely understood)."
9. **Respect Perception**: "Evaluate the extent to which the patient feels respected by the therapist. Rate from 0 (no respect) to 100 (full respect)."
10. **Emotional Bond**: "Please rate the strength of the emotional bond between the patient and the therapist. Rate from 0 (no bond) to 100 (very strong bond)."

These prompts aim to capture various facets of client motivation and the quality of the therapeutic relationship, which are crucial for understanding engagement in psychotherapy.

Excellent! Thank you! To get an idea of what words are associated with engagement, can you please list 20 words similar or synonyms to "engaged", for example "involved", "motivated", "participating", "committed", "giving effort", "showing initiative", etc.

Sure, here are 20 words that are similar or synonymous to "engaged" in the context of psychotherapy and client involvement:

1. **Involved**
2. **Motivated**
3. **Participating**
4. **Committed**
5. **Invested**
6. **Dedicated**
7. **Active**
8. **Focused**
9. **Attentive**
10. **Proactive**
11. **Enthusiastic**
12. **Responsive**
13. **Dedicated**
14. **Eager**
15. **Immersed**
16. **Collaborative**
17. **Receptive**
18. **Interactive**
19. **Contributing**
20. **Attuned**

These terms reflect various aspects of engagement, such as active participation, motivation, commitment, and proactive involvement in the therapeutic process.

Do you have 20 more please?

Sure, here are 20 more words similar or synonymous to "engaged" in the context of psychotherapy and client involvement:

1. **Absorbed**
2. **Committed**
3. **Concentrated**
4. **Curious**
5. **Driven**
6. **Embraced**

7. **Energetic**
8. **Engrossed**
9. **Industrious**
10. **Initiating**
11. **Intense**
12. **Keen**
13. **Persistent**
14. **Purposeful**
15. **Ready**
16. **Steadfast**
17. **Tenacious**
18. **Vigilant**
19. **Willing**
20. **Zealous**

These additional terms capture further nuances of engagement, including a strong interest, energy, and persistence in the therapeutic process.

Are there any other words that I should know or associate with engagement or do you think these are pretty much the most important words?

The lists provided cover a broad range of words that describe various aspects of engagement in the context of psychotherapy. However, to ensure comprehensiveness, here are a few additional words and phrases that can be associated with engagement:

1. **Activated**
2. **Aligned**
3. **Attentive**
4. **Determined**
5. **Devoted**
6. **Diligent**
7. **Engrossed**
8. **Enthralled**
9. **In-tune**
10. **Interested**
11. **Mindful**
12. **Motivated**
13. **Occupied**
14. **Plugged-in**
15. **Present**
16. **Prompt**

17. **Ready**
18. **Responsive**
19. **Steadfast**
20. **Vested**

These additional terms help to further articulate the nuances of engagement, highlighting various dimensions such as mental involvement, emotional investment, readiness, and proactive behavior. Together with the previous lists, they should provide a comprehensive vocabulary for discussing client engagement in psychotherapy.

Anything else or is it pretty much it?

The lists provided so far encompass a comprehensive range of words associated with engagement in the context of psychotherapy. These terms capture various facets such as active participation, commitment, motivation, and emotional investment. However, to ensure you have a full spectrum of terms, here are a few more that might be useful:

1. **Alert**
2. **Ardent**
3. **Assiduous**
4. **Connected**
5. **Dedicated**
6. **Devoted**
7. **Energized**
8. **Entranced**
9. **Focused**
10. **Interactive**
11. **Involved**
12. **Observant**
13. **Onboard**
14. **Passionate**
15. **Perceptive**
16. **Persistent**
17. **Present**
18. **Responsive**
19. **Single-minded**
20. **Studious**

These additional terms help to describe engagement from various angles, ensuring you have a rich vocabulary to draw from when assessing and discussing client engagement in psychotherapy.

Overall, this extensive collection of words should cover most aspects of engagement. If you need more specific terms or nuances, it might be beneficial to look at the specific context or behaviors you are trying to describe.

Perfect, thank you! If you had to choose the 50 best words representing "engaged", which ones would you choose?

Certainly! Based on the variety of contexts in which the term "engaged" can be relevant in psychotherapy, here are 50 words that encapsulate the essence of engagement, emphasizing active involvement, emotional investment, cognitive attention, and commitment:

1. **Involved**
2. **Motivated**
3. **Participating**
4. **Committed**
5. **Invested**
6. **Dedicated**
7. **Active**
8. **Focused**
9. **Attentive**
10. **Proactive**
11. **Enthusiastic**
12. **Responsive**
13. **Eager**
14. **Immersed**
15. **Collaborative**
16. **Receptive**
17. **Interactive**
18. **Contributing**
19. **Attuned**
20. **Absorbed**
21. **Driven**
22. **Engrossed**
23. **Industrious**
24. **Initiating**
25. **Intense**
26. **Keen**
27. **Persistent**
28. **Purposeful**
29. **Ready**
30. **Steadfast**

31. **Tenacious**
32. **Vigilant**
33. **Willing**
34. **Zealous**
35. **Activated**
36. **Aligned**
37. **Determined**
38. **Devoted**
39. **Diligent**
40. **Enthralled**
41. **In-tune**
42. **Interested**
43. **Mindful**
44. **Occupied**
45. **Plugged-in**
46. **Present**
47. **Prompt**
48. **Single-minded**
49. **Studious**
50. **Vested**

These words reflect a range of behaviors and attitudes that signify deep engagement in psychotherapy, from the readiness and openness to participate to the dedication and intense focus on therapeutic processes.

Perfect, thank you! :) Can you please now help me to formulate a prompt for each "engagement" word, for example: Please rate how absorbed the patient is by the therapy process on a scale from 0 (not absorbed at all) to 100 (very much absorbed). Please rate how active the patient is in therapy on a scale from 0 (not active at all) to 100 (very active). Please rate how connected the patient is with the therapist on a scale from 0 (not connected at all) to 100 (very connected). Please rate how willing the patient is to engage in therapy on a scale from 0 (not willing at all) to 100 (highly willing to engage).

Certainly! Here are prompts formulated for each "engagement" word to assess different aspects of a patient's engagement in therapy:

1. **Absorbed**: "Please rate how absorbed the patient is by the therapy process on a scale from 0 (not absorbed at all) to 100 (very much absorbed)."
2. **Activated**: "Please rate how activated the patient feels during therapy sessions on a scale from 0 (not activated at all) to 100 (highly activated)."
3. **Active**: "Please rate how active the patient is in therapy on a scale from 0 (not active at all) to 100 (very active)."

4. **Alert**: "Please rate how alert the patient is during therapy sessions on a scale from 0 (not alert at all) to 100 (extremely alert)."
5. **Aligned**: "Please rate how aligned the patient feels with the therapy goals on a scale from 0 (not aligned at all) to 100 (perfectly aligned)."
6. **Ardent**: "Please rate how ardent the patient is about their therapeutic progress on a scale from 0 (not ardent at all) to 100 (extremely ardent)."
7. **Assiduous**: "Please rate how assiduous the patient is in following therapeutic advice on a scale from 0 (not assiduous at all) to 100 (very assiduous)."
8. **Attentive**: "Please rate how attentive the patient is during therapy sessions on a scale from 0 (not attentive at all) to 100 (highly attentive)."
9. **Attuned**: "Please rate how attuned the patient is to their emotions during therapy on a scale from 0 (not attuned at all) to 100 (fully attuned)."
10. **Aware**: "Please rate how aware the patient is of their behavioral patterns on a scale from 0 (not aware at all) to 100 (very aware)."
11. **Bonded**: "Please rate how bonded the patient feels with the therapist on a scale from 0 (not bonded at all) to 100 (strongly bonded)."
12. **Captivated**: "Please rate how captivated the patient is by the therapy discussions on a scale from 0 (not captivated at all) to 100 (fully captivated)."
13. **Collaborating**: "Please rate how well the patient collaborates with the therapist on a scale from 0 (not collaborating at all) to 100 (fully collaborating)."
14. **Collaborative**: "Please rate how collaborative the patient is in the therapeutic process on a scale from 0 (not collaborative at all) to 100 (highly collaborative)."
15. **Committed**: "Please rate how committed the patient is to the therapy process on a scale from 0 (not committed at all) to 100 (fully committed)."
16. **Communicative**: "Please rate how communicative the patient is during therapy sessions on a scale from 0 (not communicative at all) to 100 (very communicative)."
17. **Concentrated**: "Please rate how concentrated the patient is during therapy sessions on a scale from 0 (not concentrated at all) to 100 (highly concentrated)."
18. **Connected**: "Please rate how connected the patient feels to the therapist on a scale from 0 (not connected at all) to 100 (very connected)."
19. **Conscientious**: "Please rate how conscientious the patient is about applying therapeutic techniques outside of sessions on a scale from 0 (not conscientious at all) to 100 (highly conscientious)."
20. **Contributing**: "Please rate how much the patient contributes to the therapy sessions on a scale from 0 (not contributing at all) to 100 (actively contributing)."
21. **Curious**: "Please rate how curious the patient is about exploring new therapeutic ideas on a scale from 0 (not curious at all) to 100 (very curious)."
22. **Dedicated**: "Please rate how dedicated the patient is to achieving their therapy goals on a scale from 0 (not dedicated at all) to 100 (fully dedicated)."

23. **Determined**: "Please rate how determined the patient is to overcome their challenges on a scale from 0 (not determined at all) to 100 (very determined)."
24. **Devoted**: "Please rate how devoted the patient is to the therapy sessions on a scale from 0 (not devoted at all) to 100 (extremely devoted)."
25. **Diligent**: "Please rate how diligent the patient is in completing therapy homework on a scale from 0 (not diligent at all) to 100 (extremely diligent)."
26. **Driven**: "Please rate how driven the patient is towards personal growth through therapy on a scale from 0 (not driven at all) to 100 (very driven)."
27. **Dynamic**: "Please rate how dynamic the patient is in responding to changes during therapy on a scale from 0 (not dynamic at all) to 100 (highly dynamic)."
28. **Eager**: "Please rate how eager the patient is to engage in therapy sessions on a scale from 0 (not eager at all) to 100 (extremely eager)."
29. **Embraced**: "Please rate how much the patient has embraced the therapeutic methods suggested on a scale from 0 (not embraced at all) to 100 (fully embraced)."
30. **Empowered**: "Please rate how empowered the patient feels by the therapy on a scale from 0 (not empowered at all) to 100 (fully empowered)."
31. **Energetic**: "Please rate how energetic the patient appears during therapy sessions on a scale from 0 (not energetic at all) to 100 (highly energetic)."
32. **Energized**: "Please rate how energized the patient feels after therapy sessions on a scale from 0 (not energized at all) to 100 (very energized)."
33. **Engaged**: "Please rate how engaged the patient is in the therapy process on a scale from 0 (not engaged at all) to 100 (fully engaged)."
34. **Engrossed**: "Please rate how engrossed the patient is in the therapy topics on a scale from 0 (not engrossed at all) to 100 (deeply engrossed)."
35. **Enthralled**: "Please rate how enthralled the patient is with the therapeutic progress on a scale from 0 (not enthralled at all) to 100 (completely enthralled)."
36. **Enthusiastic**: "Please rate how enthusiastic the patient is about continuing therapy on a scale from 0 (not enthusiastic at all) to 100 (very enthusiastic)."
37. **Entranced**: "Please rate how entranced the patient is during deeply emotional sessions on a scale from 0 (not entranced at all) to 100 (completely entranced)."
38. **Focused**: "Please rate how focused the patient is during therapy discussions on a scale from 0 (not focused at all) to 100 (extremely focused)."
39. **Harmonious**: "Please rate how harmonious the relationship between the patient and therapist is on a scale from 0 (not harmonious at all) to 100 (perfectly harmonious)."
40. **Immersed**: "Please rate how immersed the patient is in understanding their behaviors during therapy on a scale from 0 (not immersed at all) to 100 (fully immersed)."
41. **Inclusive**: "Please rate how inclusive the patient is in considering different perspectives during therapy on a scale from 0 (not inclusive at all) to 100 (very inclusive)."
42. **Industrious**: "Please rate how industrious the patient is in working on self-improvement during therapy on a scale from 0 (not industrious at all) to 100 (extremely industrious)."

43. **Influenced**: "Please rate how influenced the patient is by the therapist's guidance on a scale from 0 (not influenced at all) to 100 (highly influenced)."
44. **Initiating**: "Please rate how often the patient initiates topics or discussions in therapy on a scale from 0 (never initiates) to 100 (always initiates)."
45. **Inquisitive**: "Please rate how inquisitive the patient is about learning more about themselves through therapy on a scale from 0 (not inquisitive at all) to 100 (highly inquisitive)."
46. **Inspired**: "Please rate how inspired the patient is by the therapy sessions on a scale from 0 (not inspired at all) to 100 (greatly inspired)."
47. **Intense**: "Please rate the intensity of the patient's involvement in therapy on a scale from 0 (not intense at all) to 100 (very intense)."
48. **Interacting**: "Please rate how often the patient interacts with the therapist during sessions on a scale from 0 (rarely interacts) to 100 (frequently interacts)."
49. **Interactive**: "Please rate how interactive the patient is in the therapeutic process on a scale from 0 (not interactive at all) to 100 (highly interactive)."
50. **Interested**: "Please rate how interested the patient is in the therapy sessions on a scale from 0 (not interested at all) to 100 (very interested)."
51. **In-tune**: "Please rate how in-tune the patient is with thewith the therapeutic goals on a scale from 0 (not in-tune at all) to 100 (perfectly in-tune)."
52. **Invested**: "Please rate how invested the patient is in the outcomes of therapy on a scale from 0 (not invested at all) to 100 (fully invested)."
53. **Involved**: "Please rate how involved the patient is in the therapy process on a scale from 0 (not involved at all) to 100 (deeply involved)."
54. **Keen**: "Please rate how keen the patient is to progress in therapy on a scale from 0 (not keen at all) to 100 (extremely keen)."
55. **Mindful**: "Please rate how mindful the patient is of their thoughts and feelings during therapy on a scale from 0 (not mindful at all) to 100 (very mindful)."
56. **Motivated**: "Please rate how motivated the patient is to engage in therapy on a scale from 0 (not motivated at all) to 100 (highly motivated)."
57. **Nurtured**: "Please rate how nurtured the patient feels by the therapeutic environment on a scale from 0 (not nurtured at all) to 100 (completely nurtured)."
58. **Observant**: "Please rate how observant the patient is of changes in their mood or behavior as a result of therapy on a scale from 0 (not observant at all) to 100 (very observant)."
59. **Occupied**: "Please rate how occupied the patient is with applying therapeutic techniques in daily life on a scale from 0 (not occupied at all) to 100 (fully occupied)."
60. **Onboard**: "Please rate how onboard the patient is with the therapist's recommendations on a scale from 0 (not onboard at all) to 100 (fully onboard)."
61. **Participating**: "Please rate how actively the patient is participating in therapy sessions on a scale from 0 (not participating at all) to 100 (actively participating)."

62. **Participatory**: "Please rate how participatory the patient is in shared decision-making during therapy on a scale from 0 (not participatory at all) to 100 (highly participatory)."
63. **Passionate**: "Please rate how passionate the patient is about their therapeutic journey on a scale from 0 (not passionate at all) to 100 (very passionate)."
64. **Perceptive**: "Please rate how perceptive the patient is to insights gained during therapy on a scale from 0 (not perceptive at all) to 100 (highly perceptive)."
65. **Persistent**: "Please rate how persistent the patient is in overcoming therapeutic challenges on a scale from 0 (not persistent at all) to 100 (extremely persistent)."
66. **Plugged-in**: "Please rate how plugged-in the patient is to following up on therapy sessions on a scale from 0 (not plugged-in at all) to 100 (completely plugged-in)."
67. **Present**: "Please rate how present the patient is during therapy sessions on a scale from 0 (not present at all) to 100 (fully present)."
68. **Proactive**: "Please rate how proactive the patient is in addressing issues during therapy on a scale from 0 (not proactive at all) to 100 (highly proactive)."
69. **Prompt**: "Please rate how prompt the patient is in responding to therapeutic interventions on a scale from 0 (not prompt at all) to 100 (always prompt)."
70. **Purposeful**: "Please rate how purposeful the patient is in pursuing therapy goals on a scale from 0 (not purposeful at all) to 100 (highly purposeful)."
71. **Ready**: "Please rate how ready the patient is to engage in each therapy session on a scale from 0 (not ready at all) to 100 (completely ready)."
72. **Receptive**: "Please rate how receptive the patient is to new therapy approaches on a scale from 0 (not receptive at all) to 100 (very receptive)."
73. **Responsive**: "Please rate how responsive the patient is to feedback during therapy on a scale from 0 (not responsive at all) to 100 (highly responsive)."
74. **Single-minded**: "Please rate how single-minded the patient is in focusing on therapy outcomes on a scale from 0 (easily distracted) to 100 (completely focused)."
75. **Steadfast**: "Please rate how steadfast the patient is in adhering to the therapy process on a scale from 0 (not steadfast at all) to 100 (unwaveringly steadfast)."
76. **Studious**: "Please rate how studious the patient is in learning from therapy sessions on a scale from 0 (not studious at all) to 100 (extremely studious)."
77. **Supportive**: "Please rate how supportive the patient is in fostering a positive therapy environment on a scale from 0 (not supportive at all) to 100 (very supportive)."
78. **Synchronized**: "Please rate how synchronized the patient is with the therapist's timing and pacing during therapy on a scale from 0 (not synchronized at all) to 100 (perfectly synchronized)."
79. **Synergistic**: "Please rate how synergistic the interaction is between the patient and therapist during therapy on a scale from 0 (not synergistic at all) to 100 (highly synergistic)."
80. **Tenacious**: "Please rate how tenacious the patient is in pursuing therapeutic goals despite difficulties on a scale from 0 (not tenacious at all) to 100 (extremely tenacious)."

81. **United**: "Please rate how united the patient feels with the therapeutic objectives on a scale from 0 (not united at all) to 100 (fully united)."
82. **Vested**: "Please rate how vested the patient is in the outcomes of their therapy on a scale from 0 (not vested at all) to 100 (deeply vested)."
83. **Vigilant**: "Please rate how vigilant the patient is in maintaining therapeutic gains on a scale from 0 (not vigilant at all) to 100 (very vigilant)."
84. **Willing**: "Please rate how willing the patient is to engage in therapy on a scale from 0 (not willing at all) to 100 (highly willing to engage)."
85. **Zealous**: "Please rate how zealous the patient is about applying therapeutic techniques on a scale from 0 (not zealous at all) to 100 (extremely zealous)."

Can you please help me assign these prompts according to the model of client engagement in psychotherapy? You are also encouraged to add prompts to better assess the different elements of the model.

Engagement Determinant Variables

Clients' Motivation

- Please rate how absorbed the patient is by the therapy process on a scale from 0 (not absorbed at all) to 100 (very much absorbed)
- Please rate how motivated the patient is to engage in therapy on a scale from 0 (not motivated at all) to 100 (highly motivated)
- Please rate how committed the patient is to the therapy process on a scale from 0 (not committed at all) to 100 (fully committed)
- Please rate how driven the patient is towards personal growth through therapy on a scale from 0 (not driven at all) to 100 (very driven)
- Please rate how determined the patient is to overcome their challenges on a scale from 0 (not determined at all) to 100 (very determined)
- Please rate how eager the patient is to engage in therapy sessions on a scale from 0 (not eager at all) to 100 (extremely eager)
- Please rate how keen the patient is to progress in therapy on a scale from 0 (not keen at all) to 100 (extremely keen)
- Please rate how purposeful the patient is in pursuing therapy goals on a scale from 0 (not purposeful at all) to 100 (highly purposeful)
- Please rate how single-minded the patient is in focusing on therapy outcomes on a scale from 0 (easily distracted) to 100 (completely focused)
- Please rate how persistent the patient is in overcoming therapeutic challenges on a scale from 0 (not persistent at all) to 100 (extremely persistent)

Therapeutic Relationship

- Please rate how connected the patient feels to the therapist on a scale from 0 (not connected at all) to 100 (very connected)
- Please rate the level of trust the patient has in the therapist on a scale from 0 (no trust) to 100 (complete trust)
- How would you rate the overall quality of the therapeutic rapport between the patient and the therapist? Rate from 0 (poor rapport) to 100 (excellent rapport)
- Assess how empathetic the patient perceives the therapist to be. Rate from 0 (not empathetic) to 100 (highly empathetic)
- Assess the level of collaboration between the patient and the therapist in setting and working towards goals. Rate from 0 (no collaboration) to 100 (high collaboration)
- To what extent does the patient feel safe and supported in the therapy sessions? Rate from 0 (not safe at all) to 100 (completely safe)
- How strong is the therapeutic alliance between the patient and the therapist? Rate from 0 (weak alliance) to 100 (strong alliance)
- Evaluate the extent to which the patient feels respected by the therapist. Rate from 0 (no respect) to 100 (full respect)
- Please rate the strength of the emotional bond between the patient and the therapist. Rate from 0 (no bond) to 100 (very strong bond)
- Please rate how communicative the patient is during therapy sessions on a scale from 0 (not communicative at all) to 100 (very communicative)

Engagement Process Variables

Clients' Minimum Effort: Attendance

- Please rate how consistent the patient is in attending therapy sessions on a scale from 0 (rarely attends) to 100 (always attends)
- Please rate how punctual the patient is for therapy sessions on a scale from 0 (never on time) to 100 (always on time)

Clients' Observable, Voluntary, Active Efforts Within Sessions: Participation or Involvement

- Please rate how active the patient is in therapy on a scale from 0 (not active at all) to 100 (very active)
- Please rate how involved the patient is in the therapy process on a scale from 0 (not involved at all) to 100 (deeply involved)
- Please rate how engaged the patient is in the therapy process on a scale from 0 (not engaged at all) to 100 (fully engaged)
- Please rate how focused the patient is during therapy discussions on a scale from 0 (not focused at all) to 100 (extremely focused)

- Please rate how communicative the patient is during therapy sessions on a scale from 0 (not communicative at all) to 100 (very communicative)
- Please rate how attentive the patient is during therapy sessions on a scale from 0 (not attentive at all) to 100 (highly attentive)
- Please rate how interactive the patient is in the therapeutic process on a scale from 0 (not interactive at all) to 100 (highly interactive)
- Please rate how much the patient contributes to the therapy sessions on a scale from 0 (not contributing at all) to 100 (actively contributing)
- Please rate how actively the patient is participating in therapy sessions on a scale from 0 (not participating at all) to 100 (actively participating)
- Please rate how engrossed the patient is in the therapy topics on a scale from 0 (not engrossed at all) to 100 (deeply engrossed)

Clients' Voluntary, Active Efforts Between Sessions: Homework or Practice

- Please rate how diligent the patient is in completing therapy homework on a scale from 0 (not diligent at all) to 100 (extremely diligent)
- Please rate how often the patient applies therapeutic techniques outside of sessions on a scale from 0 (never) to 100 (always)
- Please rate how much effort the patient puts into therapeutic activities between sessions on a scale from 0 (no effort) to 100 (maximum effort)
- Please rate how often the patient reflects on therapy sessions outside of scheduled times on a scale from 0 (never) to 100 (always)
- Please rate how engaged the patient is in completing therapy homework on a scale from 0 (not engaged at all) to 100 (fully engaged)
- Please rate how proactive the patient is in following up on therapy sessions on a scale from 0 (not proactive at all) to 100 (highly proactive)
- Please rate how well the patient implements the strategies discussed in therapy on a scale from 0 (not at all) to 100 (very well)
- Please rate how much initiative the patient shows in applying what they learn in therapy to their daily life on a scale from 0 (no initiative) to 100 (high initiative)
- Please rate how motivated the patient is to complete their therapy homework on a scale from 0 (not motivated at all) to 100 (highly motivated)
- Please rate how accountable the patient is for their homework and practice activities on a scale from 0 (not accountable at all) to 100 (fully accountable)

Feel free to copy and adapt these prompts as needed for your specific purposes.
